# New benzimidazole derivatives containing hydrazone group as anticancer agents: Inhibition of carbonic anhydrase IX and molecular docking studies

**DOI:** 10.1002/ardp.202400930

**Published:** 2025-03-24

**Authors:** Hayrani Eren Bostancı, Mehmet Taha Yıldız, Serkan Kapancık, Zeynep Deniz Şahin Inan, Hacı Ahmet Kılıç, Özen Özensoy Güler, Ulviye Acar Çevik, Yusuf Özkay, Zafer Asım Kaplancıklı

**Affiliations:** ^1^ Department of Biochemistry, Faculty of Pharmacy Cumhuriyet University Sivas Turkey; ^2^ Hamidiye Faculty of Health Sciences University of Health Sciences Istanbul Turkey; ^3^ Department of Biochemistry, Faculty of Medicine Cumhuriyet University Sivas Turkey; ^4^ Department of Histology and Embryology Sivas Cumhuriyet University Sivas Turkey; ^5^ Department of Medical Biology, Faculty of Medicine Ankara Yildirim Beyazit University Ankara Turkey; ^6^ Department of Pharmaceutical Chemistry, Faculty of Pharmacy Anadolu University Eskişehir Turkey; ^7^ Medicinal Plant, Drug and Scientific Research and Application Center (AUBIBAM) Anadolu University Eskisehir Turkey; ^8^ The Rectorate of Bilecik Şeyh Edebali University Bilecik Turkey

**Keywords:** benzimidazole, carbonic anhydrase IX, flow cytometry, molecular docking

## Abstract

In this study, we propose identifying potential novel compounds targeting carbonic anhydrase (CA) IX and anticancer activity. To study the impact of these synthesized compounds on CA IX and anticancer activity, we have developed and synthesized novel benzimidazole‐hydrazone derivatives (**3a–3j**). The target compounds' ^1^H NMR (nuclear magnetic resonance), ^13^C NMR, and high resolution mass spectrometry spectra were used to confirm their chemical structures. L929 (healthy mouse fibroblast cell line) used as control healthy cell line and MCF‐7 (breast cancer), C6 (rat glioblastoma), HT‐29 (colon cancer), cells were used in cell culture studies. As a result of cell culture studies, it was determined that the newly synthesized compounds **3d** and **3j** had cytotoxic effects on colon cancer. Again, it was determined that the compound **3d** had a more toxic effect than cisplatin on both breast cancer and glioma cells. According to the CA IX activity results, compounds **3d** and **3j** were found to have the highest activity. Compounds **3d** and **3j** are essential for having anti‐cancer properties and inhibiting the carbonic anhydrase IX enzyme. Molecular docking of these compounds was carried out in the active site of CA IX. Flow cytometry and immunofluorescence microscope analyses also confirmed that these compounds had cytotoxic effects on cancer cells.

## Graphical abstract

New benzimidazole‐hydrazone derivatives (**3a–3j**) were designed and synthesized to investigate their effects on carbonic anhydrase (CA) IX and their anticancer activity. Compounds **3d** and **3j** were found to have the highest activity on CA IX and also showed anticancer properties.

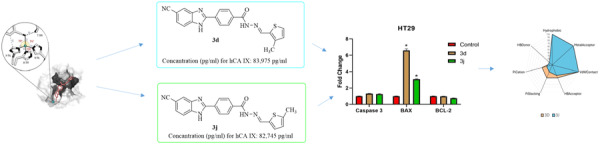

## 1 INTRODUCTION

Hypoxia is a characteristic pathological feature of the majority of solid tumors, which are the most popular forms of cancer characterized by a high level of mortality. Excessive cell division and solid tumor growth that outpaces oxygen availability lead to hypoxia. Notably, hypoxia linked to cancer can trigger angiogenesis, heighten invasiveness, aggression, and metastasis, boosting tumor survival and decreasing the beneficial effects of anticancer medications. Moreover, the reduced availability of oxygen limits the potential of oxidative phosphorylation as an energy‐producing pathway. In response to the stress of their microenvironment, hypoxic cancer cells modify their metabolism to employ the glycolytic pathway, which is far less efficient but does not require the presence of oxygen. As a result, the cell exports a high concentration of acidic metabolites, lowering the extracellular pH and increasing cancer cells' survival relative to healthy ones. Tumor growth, metastasis, invasion, and other aggressive features can be provoked by hypoxia‐induced metabolic changes and a dysregulated acid–base balance. Notably, one of the most crucial pH regulation systems necessary for cancer cell survival is carbonic anhydrase enzymes. In cancer cells, where it directly interacts with the pH regulating system, particularly bicarbonate transport metabolons, CA IX is highly expressed whereas it is expressed less in normal cells. Because of this special quality, anti‐hypoxia‐based cancer therapies can specifically target CA IX. Thus, CA‐IX is essential for controlling the pH levels within and outside of cells, as a result, it promotes the growth, progression, and metastasis of different types of tumors. Thus, the development of effective and targeted anticancer drugs can be achieved by specifically blocking the activity of CA in such conditions.^[^ ^1^, ^2^, ^3^, ^4^, ^5^ ^]^

Since heterocyclic rings are included in the architectures of many naturally occurring products and are a component of many biologically active chemicals, organic chemists should focus on synthesizing heterocyclic molecules. One of the fundamental organic elements used in the synthesis of various organic compounds, including pharmaceuticals, is a heterocyclic compound. As a result, pharmacology uses many novel medications containing heterocyclic compounds each year to treat a wide range of human illnesses. Owing to the presence of hetero atoms and the consequent wide range of characteristics, heterocyclic compounds are among the most difficult groups in chemistry.^[^ ^6^ ^]^ In medicinal chemistry, the benzimidazole scaffold—a significant member of the heterocyclic chemical class—has been the subject of extensive research. It is created when imidazole and benzene rings combine. This ring's acidic and basic NH groups demonstrate its amphoteric nature.^[^ ^7^, ^8^ ^]^ Numerous biological activities, including antimicrobial,^[^ ^9^ ^]^ antidiabetic,^[^ ^10^ ^]^ anticancer,^[^ ^11^, ^12^ ^]^ antihistaminic,^[^ ^13^ ^]^ and antihypertensive^[^ ^14^ ^]^ have been demonstrated for benzimidazole derivatives. Moreover, the benzimidazole moiety serves as the fundamental chemical framework for several commonly used drugs, such as carbendazim,^[^ ^15^ ^]^ telmisartan,^[^ ^16^ ^]^ albendazole,^[^ ^17^ ^]^ and astemizole.^[^ ^18^ ^]^ For this reason, there is still much active research in the drug design field regarding the production and biological assessment of benzimidazole molecules.

We designed and synthesized a novel series of benzimidazole‐hydrazone compounds using the justifications provided above. The synthesized compounds were evaluated for their in vitro anticancer activity, CA IX inhibition assay. Furthermore, flow cytometry and immunofluorescence microscope analyses were performed for the target compounds. Additionally, molecular docking experiments were carried out at the active region to identify the likely binding interactions.

## 2 RESULTS AND DISCUSSION

### 2.1 Chemistry

**Scheme 1:**
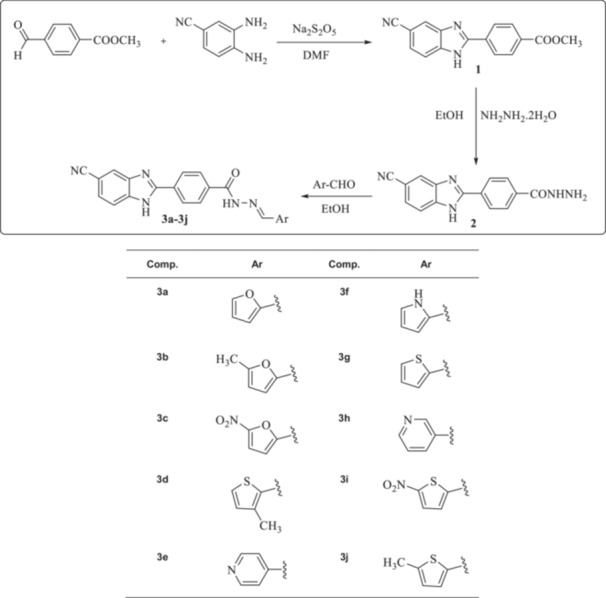
Synthesis stages of target compounds (**3a–3j**).

Synthesis of the target compounds is outlined in Scheme 1. In this study, 10 new compounds bearing the benzimidazole‐hydrazone structure were synthesized and their structures were elucidated by spectroscopic methods (^1^H NMR [nuclear magnetic resonance], ^13^C NMR, and high resolution mass spectrometry [HRMS]). In the first step of the synthesis studies, methyl 4‐formylbenzoate and sodium metabisulfide were reacted in dimethylformamide under microwave irradiation, and because of the condensation reaction of the resulting benzaldehyde sodium metabisulfite adduct and 5‐cyano 3,4‐diamino benzoate under microwave irradiation, compound **1** derivative was obtained.^[^ ^19^ ^]^ In the next step, compound **1** was treated with hydrazine hydrate under microwave irradiation to obtain 4‐(5(6)‐cyano‐1*H*‐benzimidazol‐2‐yl)benzoic acid hydrazide (**2**).^[^ ^20^ ^]^ Compound **2** and appropriate aldehyde derivatives in ethanol were refluxed and the precipitated product was filtered (**3a–3j**).

### 2.2 Pharmacology/Biology

#### 2.2.1 Anticancer activity

The compounds were examined for their preliminary anticancer activity against four different types of cell lines, such as MCF7, C6, HT29, and L929, using the 3‐[4,5‐dimethylthiazol‐2‐yl]‐2,5 diphenyl tetrazolium bromide (MTT) assay, and the obtained results were summarized in Table 1. Cisplatin was used as the positive control. As a result of the MTT study, it was observed that compounds **3a**, **3b**, **3c**, **3d,** and **3j** had a toxic effect on cancer cells. When IC_50_ values were compared, in the later stages of the study, compounds that had less toxic effects on healthy cells and had lower IC_50_ values than the positive control cisplatin were selected and immunostaining, Annexin V, cell cycle, and reverse transcription polymerase chain reaction (RT‐PCR) studies were performed. Compound **3j** was found to be effective against colon cancer, while compound **3d** was found to be effective against all three cancer cell lines (HT29, MCF7, and C6).

**Table 1:** In vitro cytotoxicity activity of **3a–3j** with IC_50_ in μM.

	L929	HT29	MCF7	C6
Control	>100	>100	>100	>100
**3a**	84.6 ± 9.6	71.8 ± 3.68	86.4 ± 1.28	98.94 ± 7.94
**3b**	>100	89.6 ± 5.74	88.4 ± 2.18	>100
**3c**	>100	90.6 ± 12.06	>100	>100
**3d**	>100	39.1 ± 8.28	25.6 ± 16.44	20.48 ± 8.34
**3e**	>100	>100	>100	>100
**3f**	>100	>100	>100	>100
**3g**	>100	>100	>100	>100
**3h**	>100	>100	>100	>100
**3i**	>100	>100	>100	>100
**3j**	>100	45.8 ± 15.58	>100	>100
Cisplatin	88.8 ± 6.4	59.4 ± 12.6	29.6 ± 7.6	39.4 ± 6.4

When the structures of the compounds are examined, it is seen that they are derivatized using different heterocyclic rings. When the anticancer activity result is evaluated, it is seen that the presence of the thiophene ring carrying the methyl substituent in the structure increases the activity. When compounds **3b** and **3j** were examined against the HT29 cell line, the activity was almost doubled with the replacement of the furan ring with the thiophene ring. In particular, compound **3j** has selective activity against the HT29 cell line. It was found that the compound **3d** carrying a methyl group in position *ortho* was also effective in all cancer cell lines. Activity decreases with the substitution of nitro, an electron‐withdrawing group, on the thiophene ring.

#### 2.2.2 Flow cytometry

**Figure 1:**
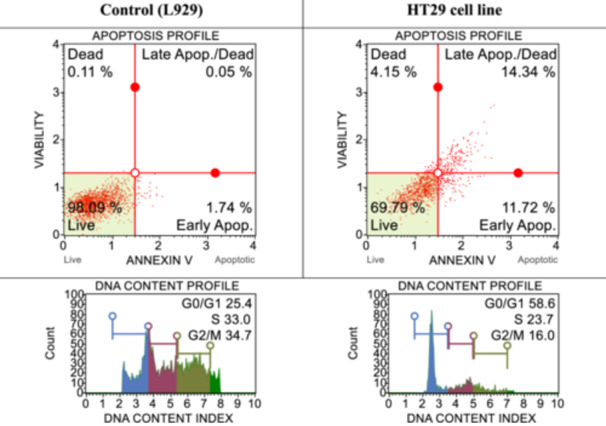
After 24 h of incubation, apoptotic and cell‐cycle consequences of compound **3d** on L929 and HT29 with IC_50_ dose.

**Figure 2:**
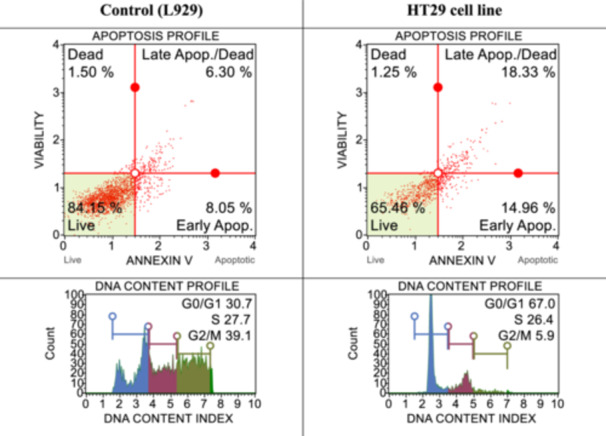
After 24 h of incubation, apoptotic and cell‐cycle consequences of compound **3j** on L929 and HT29 with IC_50_ dose (45.8 ± 15.58 µM).

**Figure 3:**
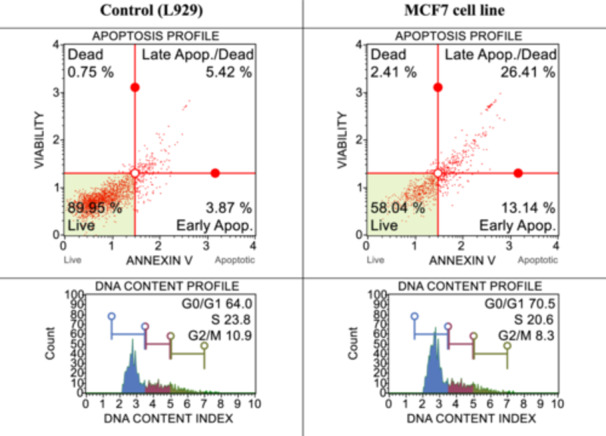
After 24 h of incubation, apoptotic and cell‐cycle consequences of compound **3d** on L929 and MCF7 with IC_50_ dose (25.6 ± 16.44 µM).

**Figure 4:**
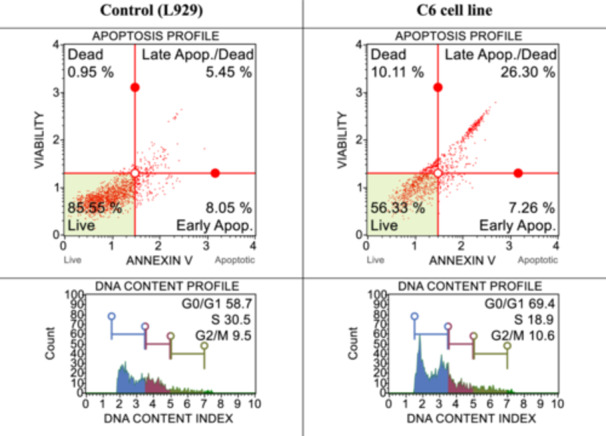
After 24 h of incubation, apoptotic and cell‐cycle consequences of compound **3d** on L929 and C6 with IC_50_ dose (20.48 ± 8.34 µM).

With the flow cytometry studies described previously,^[^ ^21^ ^]^ cell death pathways and cell‐cycle studies were performed in both healthy cells and determined cancer cells by applying IC_50_ doses of syntheses determined to be effective for cancer cells. The results obtained are shown in Figures 1, 2, 3, 4. When the results are examined, the number of live cells in the control group cells is higher than in the cancer cells. At the same time, the percentage of cells undergoing apoptosis in the cancer cells is much higher than the percentage of dead (necrotic) cells. When the cell cycles are examined, the cancer cells are observed to terminate the cycle in the G0/G1 phase of their division phase.

#### 2.2.3 Immunofluorescent microscope analysis

The well‐known proliferation marker Ki67 is used to assess cell proliferation. Numerous studies have shown that increased Ki67 localization in cells is blunted by cancer progression. Although Ki67 is almost undetectable in normal cells, it has become a promising target for cancer therapy because it is highly expressed in malignant cells.^[^ ^22^ ^]^

**Figure 5:**
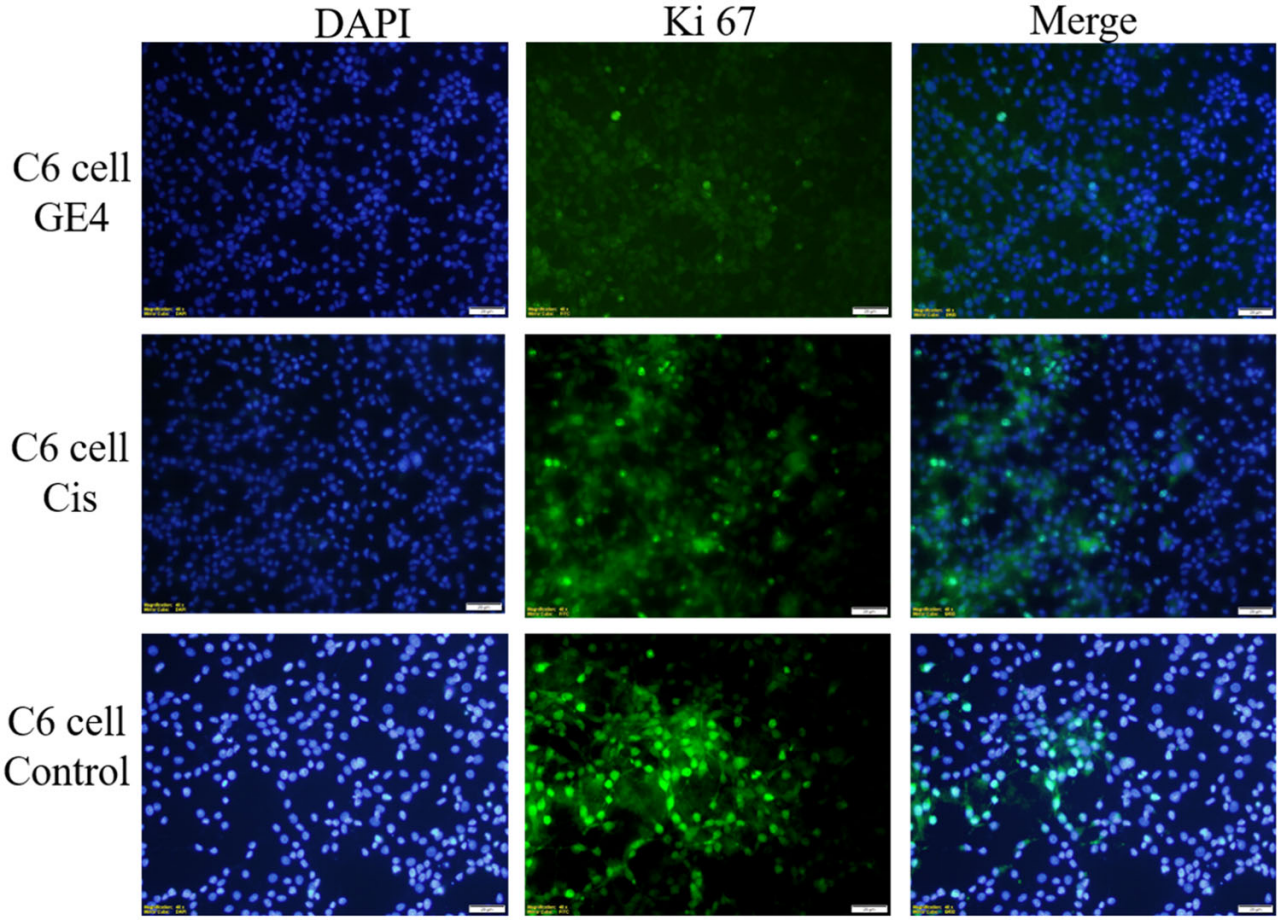
Compound **3d** (GE4), cisplatin (cis), and Ki67 positive cells (green) in the C6 cell line grown in medium; whole cell nuclei are shown with Dapi (blue) staining, images are merged (×40 magnification, OlympusBX51).

**Figure 6:**
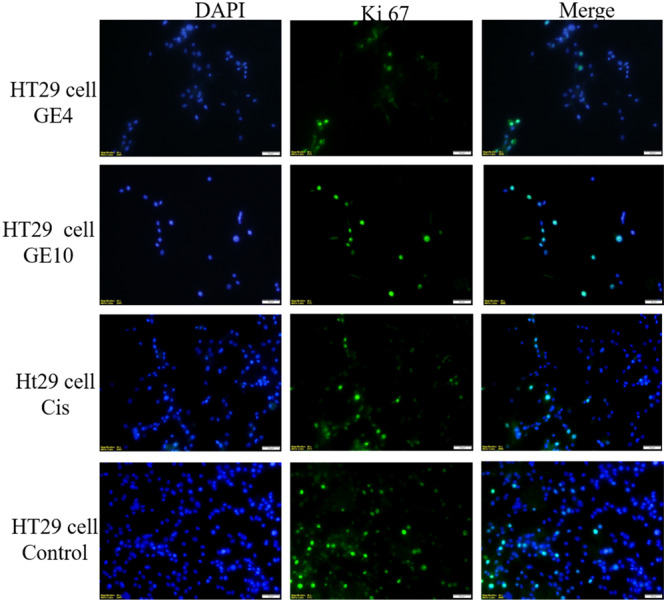
Compound **3d** (GE4), compound **3j** (GE10), cisplatin (cis), and Ki67 positive cells (green) in the HT29 cell line grown in medium; all cell nuclei are shown with Dapi (blue) staining, images are merged (×40 magnification, OlympusBX51).

**Figure 7:**
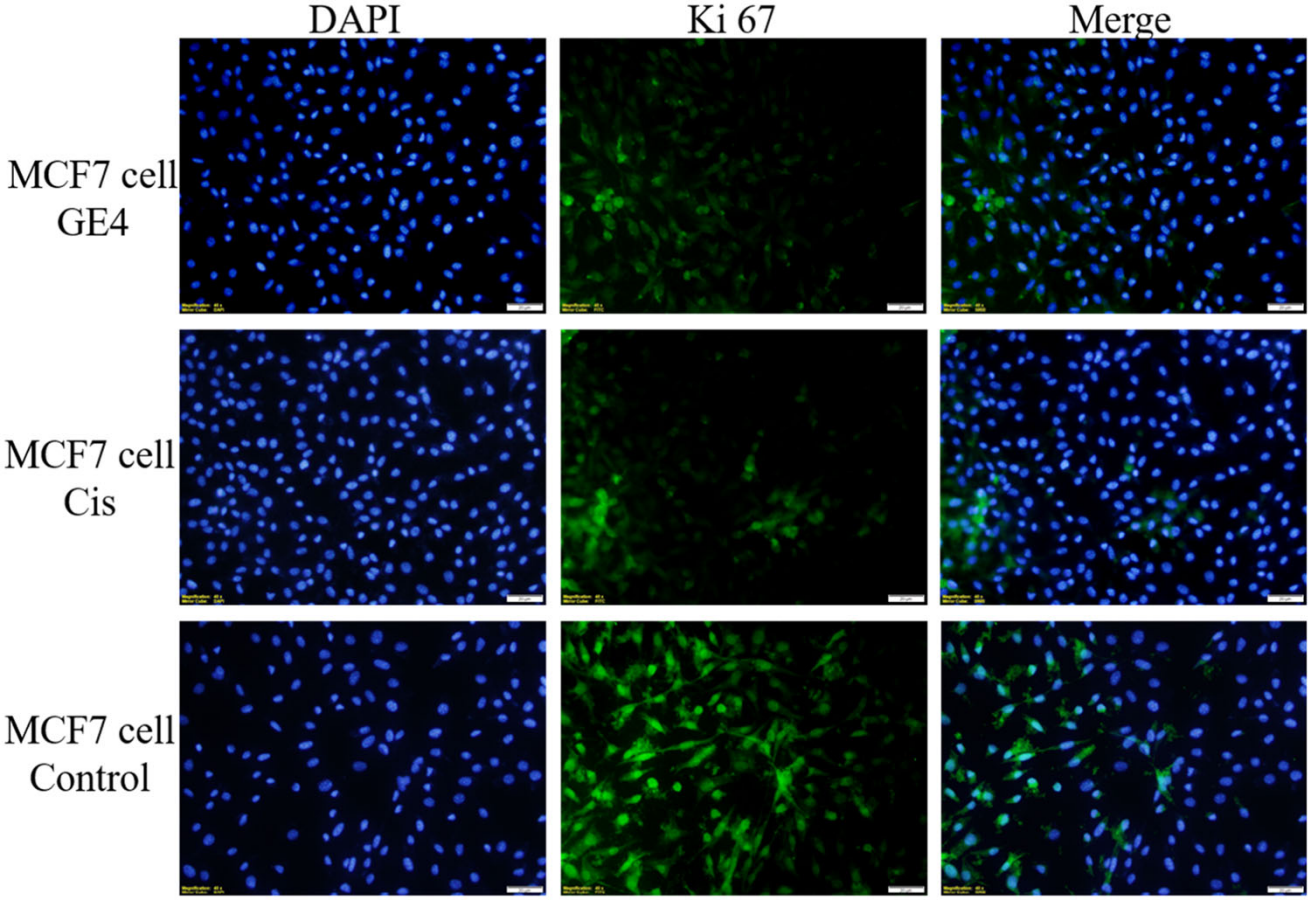
Compound **3d** (GE4), cisplatin (cis), and Ki67 positive cells (green) in MCF7 cell line grown in medium; whole cell nuclei are shown with Dapi (blue) staining, images are merged (×40 magnification, OlympusBX51).

**Figure 8:**
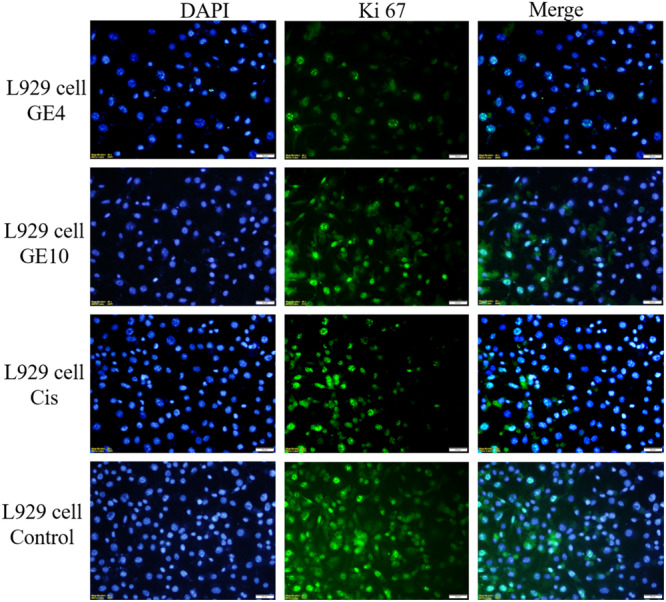
Compound **3d** (GE4), compound **3j** (GE10), cisplatin (cis), and Ki67 positive cells (green) in the L929 cell line grown in medium; all cell nuclei are shown with Dapi (blue) staining, images are merged (×40 magnification, OlympusBX51).

Proliferating cells are the only ones that express the nuclear Ki67 protein. During interphase, Ki67 is mostly located in the nucleolar cortex, and during mitosis, it is drawn to condensed chromosomes. The two Ki67 isoforms, 345 and 395 kDa, are encoded by the Ki67 gene, which is found on chromosome 10q25‐ter. From G1 phase to mitosis, the level of Ki67 expression rises, and it quickly falls following mitosis. The nuclei of cells in the G1, S, G2, and mitotic phases all contain Ki67 protein, whereas the nuclei of quiescent cells in the G0 phase do not. Thus, the level of Ki67 expression reflects the stage of cell division. Indeed, Ki67 has been suggested as a predictive indicator of cancer and is significantly overexpressed in cancer cells.^[^ ^22^ ^]^ The anticancer activity of the compound applied on cells with a high proliferation rate in immunofluorescence staining is shown by decreased Ki67 expression.^[^ ^22^, ^23^ ^]^ A study in adult wild‐type glioblastoma cells demonstrates a potential relationship between Ki67 index and morphological biomarkers for proliferation.^[^ ^24^ ^]^ In this study, Ki67 immunolocalisation rate was significantly decreased in compound **3d** applied on C6 and MCF7 cell lines, compounds **3d** and **3j** in HT29 and L929 cell lines compared with the cisplatin‐treated group. In HT29 and L929 cell lines, compound **3d** causes a more significant decrease in Ki67 positive cells than compound **3j** (Figures 5, 6, 7, 8 **)**.

#### 2.2.4 RT‐PCR

**Figure 9:**
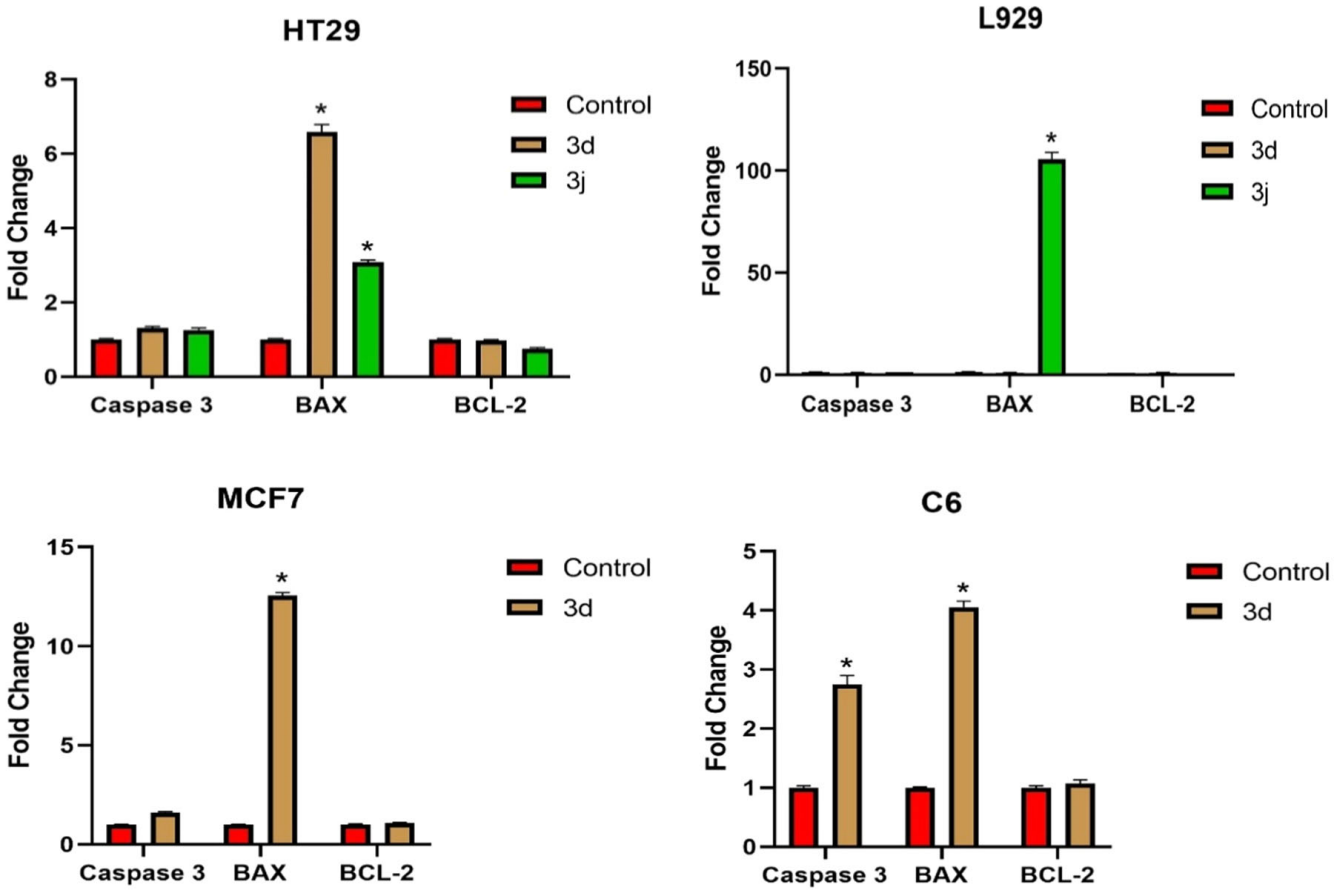
Caspase 3, BAX, and BCL‐2 expression profiles in cells treated with effective doses of compounds **3d** and/or **3j**.

Determination of Caspase 3, B‐cell lymphoma gene‐2 (Bcl‐2)‐associated X protein (BAX), and BCL‐2 expression profiles in cells applied effective doses of **3d** and/or **3j**:

Caspase 3, BAX, and BCL‐2 expression levels were detected in cDNA samples obtained from cells treated with effective **3d** and/or **3j** doses for 24 h and control group cell lines (Figure 9). It was determined that BAX increased significantly by 6.59 times in HT29 cell lines exposed to the effective dose of compound **3d**, and BAX increased significantly by 3.07 times in HT29 cell lines exposed to compound **3j** (*p* < 0.05). It was determined that BAX increased significantly by 105.7 times in L929 cell lines exposed to the effective dose of compound **3j** (*p* < 0.05). In MCF7 cell lines exposed to the effective dose of compound **3d**, BAX was found to increase significantly by 12.55 times (*p* <0.05). It was determined that Caspase 3 increased significantly by 2.75 times and BAX by 4.06 times in C6 cell lines exposed to the effective dose of compound **3d** (*p* <0.05).

Our study found that apoptotic deaths in cells applied to adequate doses of compounds **3d** and/or **3j** for 24 h increased significantly. It has been determined that the induction of cell apoptosis following exposure to compounds **3d** and/or **3j** may be related to the increase in Caspase 3 and BAX, genes related to apoptosis.

In its synthetic form, caspase 3 is a 32‐kDa proenzyme.^[^ ^25^ ^]^ Two 12‐kDa and 17‐kDa subunits combine to create the active Caspase 3 enzyme when the inactive Caspase 3 enzyme is split into 12‐kDa and 17‐kDa subunits. Caspase 3 not only breaks down proteins involved in many cell structures but also causes DNA breaks, activating DNAse and thus apoptosis. The fact that compound **3d** application causes an increase in Caspase 3 levels in cells indicates that compound **3d** induces apoptosis in cells by increasing Caspase 3 expression.

One of the apoptosis regulators, the BAX protein, causes apoptosis, particularly when mitochondrial diseases occur. When BAX is activated, mitochondria permeability increases, followed by the release of cytochrome c and cellular apoptosis. It is well known that by activating BAX, certain medications used to treat cancer aid in its therapy. The increase in BAX expression due to exposure to compounds **3d** and/or **3j** will induce apoptosis by causing an increase in the cell mitochondria permeability. This indicates that **3d** and/or **3j** series drugs also increase apoptosis by mediating an increase in mitochondria permeability.

#### 2.2.5 CA IX inhibition assay

**Figure 10:**
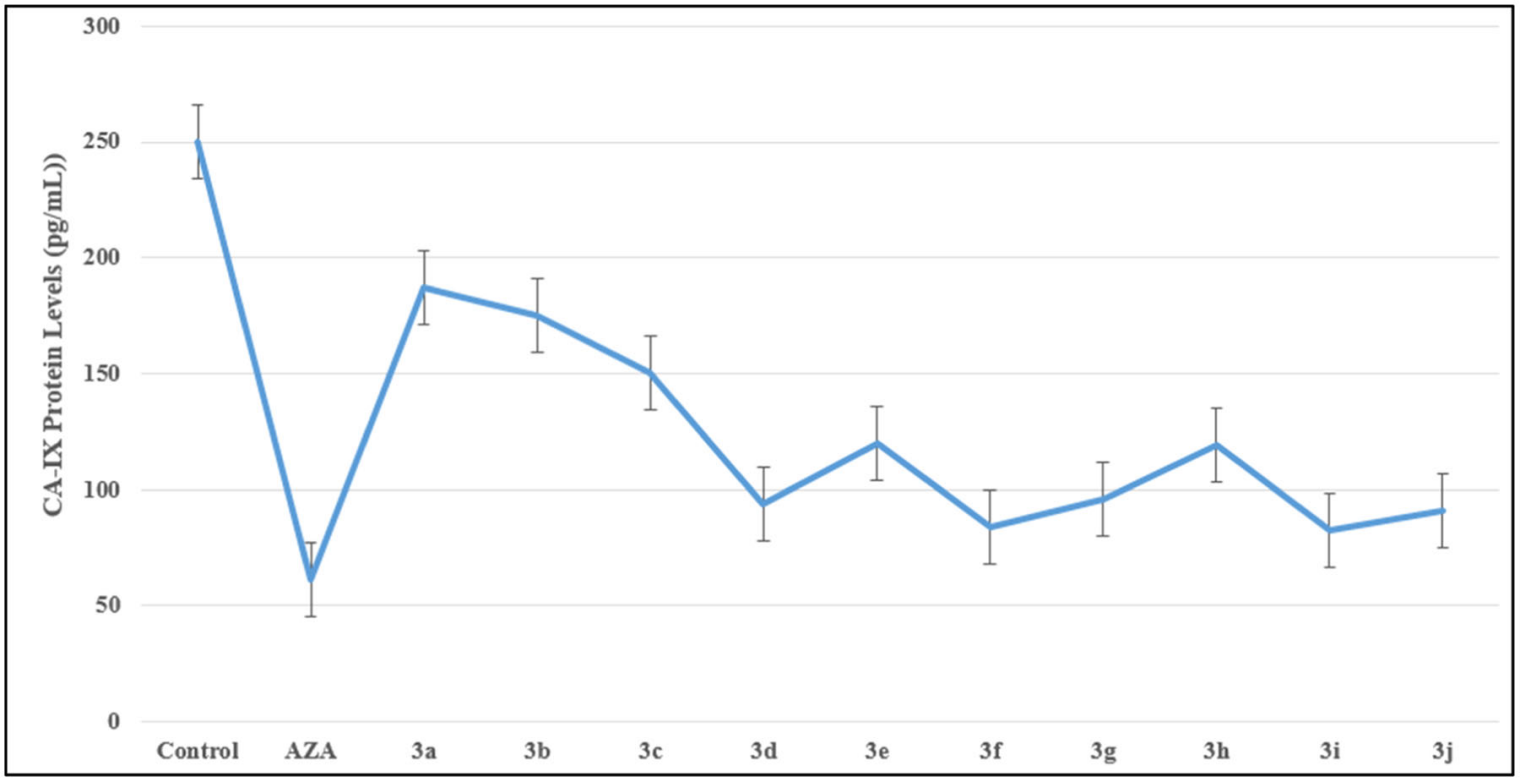
Carbonic anhydrase IX (CA‐IX) protein level (**3a–j**) (*p* < 0.001).

All synthesized compounds (**3a–3j**) were evaluated for their inhibitory potency against CA IX enzyme using ELISA Kit. The CA IX inhibitor acetazolamide was used as a positive control. The CA IX inhibitory activity of the synthesized compounds (**3a–3j**) was shown as concentration and summarized in Table 2 and Figure 10. Among them, compounds **3d** and **3j** have the strongest inhibition value. Compound **3f** with a 5‐methylthiophen substituent showed lower CA IX inhibition concentration than compound **3d** with a 3‐methylthiophen substituent. Compounds **3f**, **3g**, and **3i** have moderate inhibitory potential. When the activities of the compounds were evaluated, it was seen that the compounds carrying thiophene ring have higher CA IX inhibition potential.

**Table 2:** ELISA experiment for series **3a–j** (*p* < 0.001).

Compound	Concentration (pg/mL)
Control	250,225
**Acetazolamide**	60,945
**3a**	186,945
**3b**	175,225
**3c**	150,225
**3d**	83,975
**3e**	120,225
**3f**	93,975
**3g**	95,745
**3h**	119,425
**3i**	92,225
**3j**	82,745

Abbreviation: ELISA, enzyme‐linked immunosorbent assay.

#### 2.2.6 Molecular docking

One of the essential features an ideal inhibitor should possess is a functional zinc binding group (ZBG) capable of coordinating with the Zn (II) ion located at the base of the active site, approximately 15 Å deep from the surface. Well‐known ZBGs, such as sulfonamides, sulfamates, and carboxylates, typically coordinate with the zinc ion or the zinc‐bound water/hydroxide ion.^[^ ^26^, ^27^, ^28^, ^29^, ^30^ ^]^ While there are exceptions, the second key structural element of a traditional carbonic anhydrase inhibitor (CAI) is the scaffold, composed of aromatic and heteroaromatic rings. This scaffold can stabilize the ligand through hydrophobic and hydrophilic interactions with residues within the active site, serving as a central core that anchors the ZBG on one side, while the ‘tail’ extends on the other side.^[^ ^19^, ^30^, ^31^, ^32^ ^]^ The strategy of appending “tails” to scaffolds has been explored to create elongated molecules that interact with isoform‐specific residues in the selective pocket of the active site (Zone‐I: Gln 67; Zone‐II: Leu 91, Val 131).^[^ ^19^, ^29^, ^30^ ^]^ Since the selective pocket residues are near conserved residues, inhibitors may need to extend their interactions beyond Zone II into the peripheral Zone III to distinguish between CA isoforms effectively.^[^ ^29^ ^]^

**Figure 11:**
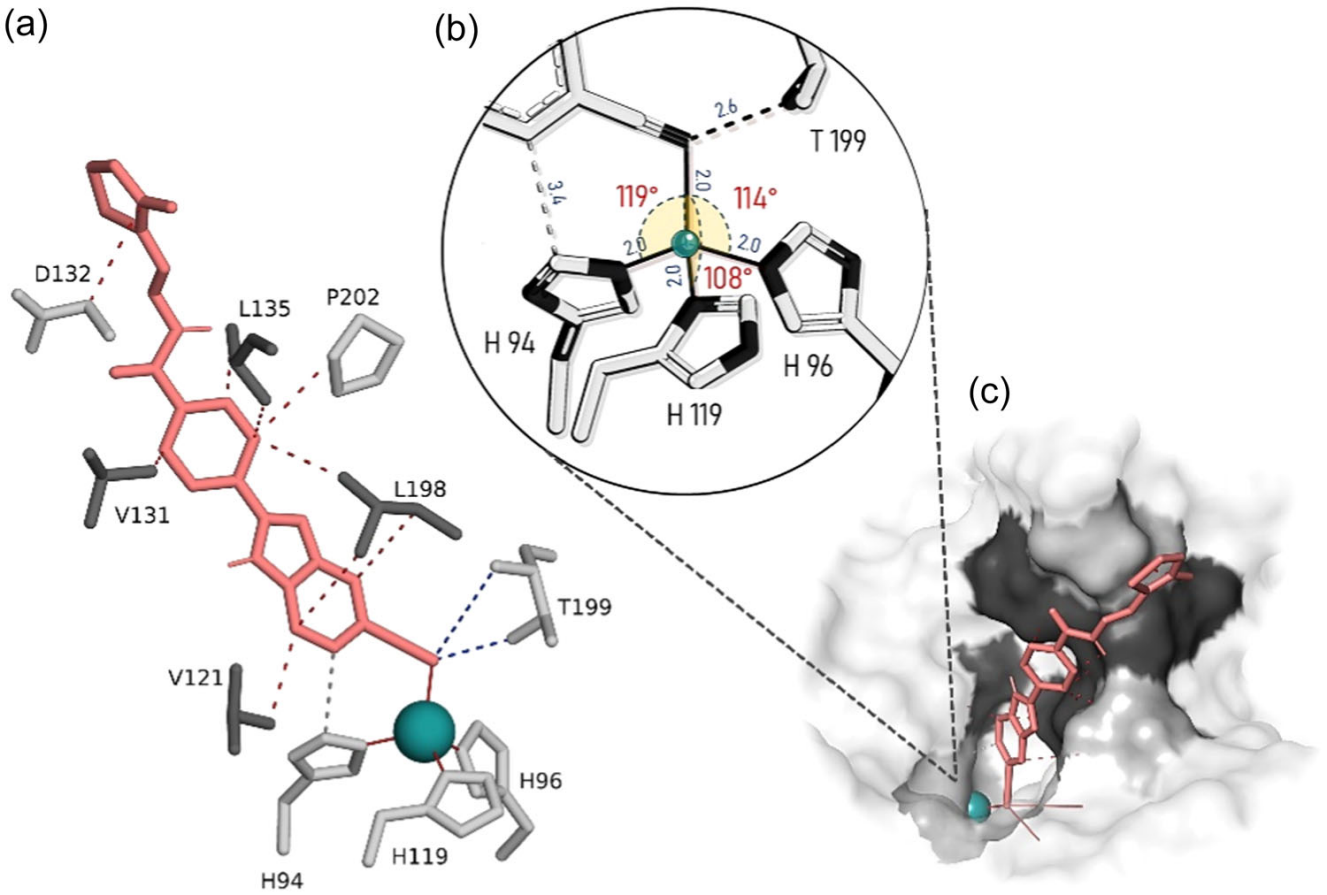
Docking Pose: (a) Compound **3d** interacting with key residues in the active site and hydrophobic rim. Dashed red lines represent hydrophobic and van der Waals interactions, dashed blue lines indicate hydrogen bonds, and solid red lines show metal coordination bonds. (b) Interactions of the ZBG with Zn(II), forming a distorted tetrahedral geometry. The angles and distances reflect the average of the first 10 energetically favorable poses. The bond angles and the cyano nitrogen‐Zn(II) distance suggest a near‐ideal geometry. In addition to metal coordination, a hydrogen bond between Thr 199 and the cyano nitrogen (dashed black line) and a hydrophobic interaction with His 94 (dashed gray line) are shown. (c) Surface representation of the same pose. Gray areas indicate hydrophilic regions, while black regions are hydrophobic. The ligand interacts with Zn (II) at the bottom of the active site (green sphere) via the zinc binding group (ZBG), while attaching itself to Zone II residues through hydrophobic interactions. The tail extends toward the outer peripheral region, interacting with hydrophobic residues in Zone III.

The analyses suggest that both ligands may coordinate with the Zn (II) ion through the cyano nitrogen of the ZBG, adopting tetrahedral geometry and establishing favorable interactions with active site residues. Previous studies have shown that the cyano group can act as a ZBG in certain contexts. However, these findings should be regarded as hypothetical until validated experimentally. These results indicate that the ligands are anchoredinto the CA core by forming a hydrogen bond with Thr199 (Figure 11) at high interaction frequencies (97%) andcoordinating with the positively charged zinc ion in the proper tetrahedralgeometry through their negatively charged cyano's nitrogen.

**Figure 12:**
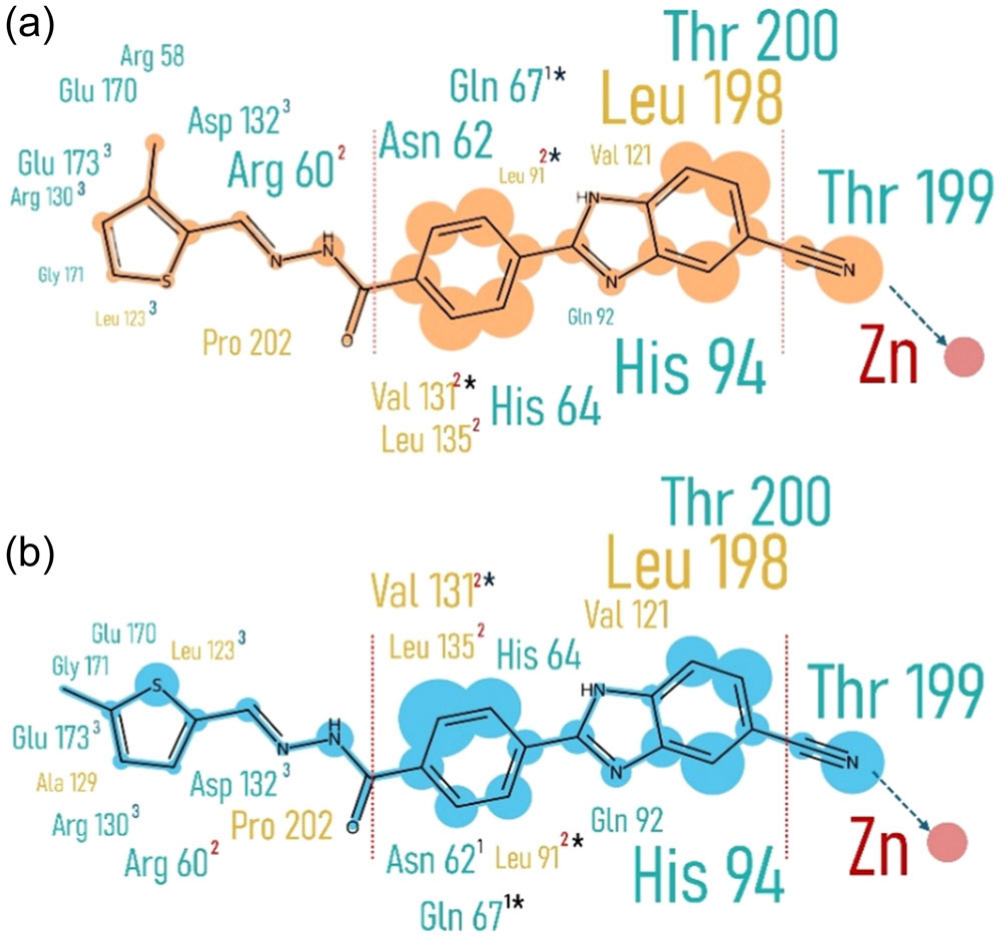
Comparison of the interaction frequencies of ligand atoms and residues: The size of the residue names and the shaded areas around the ligand atoms are proportional to their interaction frequencies, as calculated by ProLIF. Zones are represented by uppercase numbers displayed alongside the residue names, indicating the specific regions each residue belongs to. Residues within the “specific pocket” are highlighted with asterisks (*). Light brown shading denotes hydrophobic interactions, while cyan shading represents hydrophilic interactions. Panel a illustrates the interaction map for compound **3d**, while Panel b depicts the interactions for compound **3j**.

Overall, the scaffold likely interacts with the hydrophobic half of the active site cleft like benzenesulfonamides and thiadiazole derivatives, binding key residues like Gln92, Val121, Leu198, Thr199, Thr200, and Pro202.^[^ ^26^ ^]^ Docking simulation analysis further confirms that the scaffold, made of aromatic and heterocyclic rings, is the most interactive component of compounds **3d** and **3j** (Figure 12). As a result, both ligands potentially meet key criteria by coordinating with Zn(II) to form a tetrahedral geometry and interacting with the base residue Thr199 via the ZBG. The scaffold appears to establish hydrophobic interactions with residues in Zones I and II, particularly Gln67 and Val131, within the selective pocket, which are critical for inhibitory activity and span both halves of the active site rim. Additionally, the tail group interacts with peripheral Zone III residues—predominantly hydrophilic for **3d** and a mix of hydrophilic and hydrophobic for **3j**—potentially contributing to isoform selectivity.

## 3 CONCLUSION

MTT, cell death, and cell‐cycle studies performed on cancer cells demonstrated that compound **3d** is effective against various cancer types, while compound **3j** showed particular efficacy in colon cancer. Compound **3a**, although effective against cancer cells, was excluded from further studies due to its toxicity to healthy cells. Similarly, compound **3b**, despite showing toxic effects on colon and breast cancer cells, had higher IC_50_ values compared with the positive control cisplatin, leading to its exclusion from further analyses. Flow cytometry results, consistent with MTT assays, confirmed the findings of cell death and cell‐cycle studies.

The assay results highlight the strong inhibitory activity of compounds **3d** and **3j** against the CA IX enzyme and their efficacy in anticancer treatments. Both compounds exhibited significant effects in MTT assays, cell death, and cell‐cycle studies. Compound **3d** demonstrated broad‐spectrum effectiveness across various cancer cell lines, while compound **3j** exhibited potent activity specifically against colon cancer. RT‐PCR and immunostaining studies further supported the efficacy of **3d** and **3j** in targeting cancer cells.

The structural difference between **3d** and **3j**—the positioning of the CH_3_ group on the thiophene ring—likely impacts their specific interactions, particularly within Zone III. Docking studies corroborate these observations, highlighting strong interactions between these compounds and the active site of the CA IX enzyme. Compounds containing a thiophene ring, such as **3d** and **3j**, demonstrated higher potential for CA IX inhibition. This effect may stem from the stable and well‐defined interactions between the compounds’ structural components (ZBG, scaffold, and tail) and key residues in Zones II and III. These interactions are considered crucial in promoting selective binding and stabilizing the compounds within the enzyme's active site.

In summary, although further studies are needed to confirm these findings, the structural features of compounds **3d** and **3j**—particularly the incorporation of a thiophene ring and the precise positioning of substituents—could potentially play a role in their inhibitory activity against CA IX and their effectiveness in cancer cell inhibition. While structurally similar, the slightly broader interaction profile of **3j** might contribute to enhanced selectivity for CA IX, possibly reducing off‐target effects. In contrast, the more focused interactions of **3d** with specific residues might help explain its greater inhibitory potency compared with **3j**, as suggested by the assays. Docking studies support this hypothesis, suggesting strong interactions between these compounds and the active site of the CA IX enzyme. Compounds containing a thiophene ring, such as **3d** and **3j**, may demonstrate potential for CA IX inhibition, likely due to stable and well‐defined interactions between their structural components (ZBG, scaffold, and tail) and residues in Zones II and III, which are thought to be important for selective binding and stabilization within the enzyme's active site.

Although further studies are needed to confirm these findings, the structural features of **3d** and **3j**—particularly the inclusion of a thiophene ring and the precise positioning of substituents—appear to be critical determinants of their potent inhibitory activity against CA IX and their efficacy in cancer cell inhibition. Despite their structural similarity, **3j**'s broader interaction profile may enhance its selectivity for CA IX, potentially reducing off‐target effects. Meanwhile, **3d**'s more focused interactions with specific residues might explain its superior inhibitory potency relative to **3j**, as observed in the assays.

## 4 EXPERIMENTAL

### 4.1 Chemistry

#### 4.1.1 General remarks

All the chemicals employed in the synthetic procedure were purchased from Sigma‐Aldrich Chemicals (Sigma‐Aldrich Corp.) or Merck Chemicals (Merck KGaA). Melting points of the obtained compounds were determined by MP90 digital melting point apparatus (Mettler Toledo) and were uncorrected. ^1^H‐NMR, and ^13^C‐NMR spectra (see the Supporting Information) of the synthesized compounds were performed by a Bruker 400 and 100 MHz digital FT‐NMR spectrometer (Bruker Bioscience) in Dimethyl sulfoxide (DMSO)‐d_6_, respectively. Splitting patterns were designated as follows: s: singlet; d: doublet; t: triplet; m: multiplet in the NMR spectra. Coupling constants (J) were reported as Hertz. All reactions were monitored by thin‐layer chromatography (TLC) using Silica Gel 60 F254 TLC plates (Merck KGaA).

The InChI codes of the investigated compounds, together with some biological activity data, are provided as Supporting Information.

#### 4.1.2 General procedure for the synthesis of methyl 4‐(5(6)‐cyano‐1*H*‐benzimidazol‐2‐yl)benzoate (1) and 4‐(5(6)‐cyano‐1H‐benzimidazol‐2‐yl)benzoic acid hydrazide (2)

Compounds 1 and 2 were prepared using the reported method. ^[^ ^20^ ^]^

#### 4.1.3 General procedure for the synthesis of 4‐(5‐cyano‐1*H*‐benzo[d]imidazol‐2‐yl)‐*N*′‐[(substitutedheteroaryl)methylene]benzohydrazide (**3a‐3j**)

Hydrazone derivative compounds (**3a–3j**) were obtained by refluxing the hydrazide derivative compound (**2**) with aldehyde derivatives in ethanol.

4‐(5‐Cyano‐1*H*‐benzo[d]imidazol‐2‐yl)‐*N*′‐[(furan‐2‐yl)methylene]benzohydrazide (**3a**): Yield: 78%, M.P.= 171.0°C. ^1^H‐NMR (400 MHz, DMSO‐d_6_): *δ*: 6.64–6.66 (1H, m, Aromatic CH), 6.96–6.97 (1H, m, Aromatic CH), 7.62–7.63 (1H, m, Aromatik CH), 7.73 (1H, br.s., Aromatic CH), 7.86 (1H, s, Aromatic CH), 8.10 (3H, d, *J *= 8.40 Hz, Aromatik CH), 8.34 (2H, d, *J* = 7.84 Hz, Aromatik CH), 8.38 (1H, s, CH═N), 11.94 (1H, s, NH), 13.64 (1H, s, NH). ^13^C‐NMR (100 MHz, DMSO‐d_6_): *δ* = 112.72, 113.10, 113.38, 114.25, 117.76, 120.40, 124.49, 127.36, 128.85, 132.46, 135.32, 138.36, 145.77, 147.08, 149.59, 149.84, 150.92, 162.79. HRMS (*m/z*): [M+H]^+^ calcd for C_20_H_13_N_5_O_2_: 356.1142; found: 356.1137.

4‐(5‐Cyano‐1*H*‐benzo[*d*]imidazol‐2‐yl)‐*N*′‐[(5‐methylfuran‐2‐yl)methylene]benzohydrazide (**3b**): Yield: 80%, M.P. = 135.6°C. ^1^H‐NMR (400 MHz, DMSO‐d_6_): *δ*: 2.35 (3H, s, CH_3_), 6.25–6.26 (1H, m, Aromatic CH), 6.30–6.31 (1H, m, Aromatic CH), 6.82–6.83 (1H, m, Aromatik CH), 6.95–6.96 (1H, m, Aromatic CH), 8.09 (2H, d, *J* = 8.36 Hz, Aromatik CH), 8.27 (1H, s, Aromatik CH), 8.33 (2H, d, *J *= 7.80 Hz, Aromatic CH), 8.38 (1H, s, CH═N), 11.86 (1H, s, NH), 13.62 (1H, s, NH). ^13^C‐NMR (100 MHz, DMSO‐d_6_): *δ* = 13.92, 109.08, 109.60, 110.21, 116.11, 119.20, 120.40, 124.44, 127.31, 128.79, 132.38, 135.34, 138.24, 148.27, 148.29, 150.11, 155.20, 156.56, 162.71. HRMS (*m/z*): [M+H]^+^ calcd for C_21_H_15_N_5_O_2_: 370.1299; found: 370.1294.

4‐(5‐Cyano‐1*H*‐benzo[*d*]imidazol‐2‐yl)‐*N*′‐[(5‐nitrofuran‐2‐yl)methylene]benzohydrazide (**3c**): Yield: 81%, M.P. = 207.1°C. ^1^H‐NMR (400 MHz, DMSO‐d_6_): *δ*: 7.27 (1H, s, Aromatic CH), 7.60 (2H, d, *J *= 8.32 Hz, Aromatik CH), 7.76–7.77 (2H, m, Aromatik CH), 8.09 (2H, d, *J* = 7.80 Hz, Aromatik CH), 8.15 (1H, br.s., Aromatic CH), 8.33 (2H, d, *J* = 8.20 Hz, Aromatic CH), 8.40 (1H, s, CH═N), 12.33 (1H, s, NH). ^13^C‐NMR (100 MHz, DMSO‐d_6_): *δ *= 18.99, 104.81, 105.25, 115.06, 115.91, 120.22, 120.37, 123.43, 127.16, 127.38, 128.99, 132.79, 134.59, 136.23, 138.28, 152.10, 152.38, 163.08. HRMS (*m/z*): [M+H]^+^ calcd for C_20_H_12_N_6_O_4_: 401.0993; found: 401.1004.

4‐(5‐Cyano‐1*H*‐benzo[*d*]imidazol‐2‐yl)‐*N*′‐[(3‐methylthiophen‐2‐yl)methylene]benzohydrazide (**3d**): Yield: 77%, M.P. = 132.6°C. ^1^H‐NMR (400 MHz, DMSO‐d_6_): *δ*: 2.34 (3H, s, CH_3_), 6.97–6.98 (1H, m, Aromatic CH), 7.00–7.01 (1H, m, Aromatic CH), 7.57–7.58 (1H, m, Aromatic CH), 7.65–7.66 (1H, m, Aromatic CH), 8.10 (2H, d, *J * =8.40 Hz, Aromatik CH), 8.34 (2H, d, *J* = 7.88 Hz, Aromatik CH), 8.76 (1H, s, Aromatik CH), 8.83 (1H, s, Aromatik CH), 11.86 (1H, s, NH), 13.63 (1H, s, NH). ^13^C‐NMR (100 MHz, DMSO‐d_6_): *δ* = 14.05, 120.41, 127.34, 128.74, 130.33, 131.37, 131.75, 132.41, 132.57, 132.86, 135.36, 135.56, 140.73, 142.84, 143.59, 154.50, 162.40, 183.89. HRMS (*m/z*): [M+H]^+^ calcd for C_21_H_15_N_5_OS: 386.1070; found: 386.1063.

4‐(5‐Cyano‐1*H*‐benzo[*d*]imidazol‐2‐yl)‐*N*′‐[(pyridine‐4‐yl)methylene]benzohydrazide (**3e**): Yield: 78%, M.P. = 289.3°C. ^1^H‐NMR (400 MHz, DMSO‐d_6_): *δ*: 6.98–7.01 (2H, m, Aromatic CH), 7.57–7.58 (1H, m, Aromatic CH), 7.61 (1H, br.s., Aromatic CH), 7.65–7.66 (1H, m, Aromatic CH), 8.10 (2H, d, *J* = 8.40 Hz, Aromatic CH), 8.33–8.35 (3H, m, Aromatic CH), 8.76 (1H, s, Aromatic CH), 8.83 (1H, s, Aromatic CH), 11.86 (1H, s, NH), 13.63 (1H, s, NH). ^13^C‐NMR (100 MHz, DMSO‐d_6_): *δ* = 120.41, 127.34, 128.74, 130.33, 131.37, 131.75, 132.41, 132.57, 132.86, 135.36, 135.56, 140.73, 142.84, 143.59, 154.50, 162.40, 183.89. HRMS (*m/z*): [M+H]^+^ calcd for C_21_H_14_N_6_O: 367.1302; found: 367.1302.

4‐(5‐Cyano‐1*H*‐benzo[*d*]imidazol‐2‐yl)‐*N*′‐[(imidazol‐2‐yl)methylene]benzohydrazide (**3f**): Yield: 80%, M.P. = 161.1°C. ^1^H‐NMR (400 MHz, DMSO‐d_6_): *δ*: 7.64 (1H, dd, *J*_*1*_ = 1.36 Hz, *J*_*2*_ = 8.32 Hz, Aromatic CH), 7.65–7.66 (2H, m, Aromatic CH), 7.80–7.82 (1H, m, Aromatic CH), 8.16 (2H, d, *J* = 8.12 Hz, Aromatic CH), 8.38 (2H, d, *J *= 8.32 Hz, Aromatic CH), 8.51 (1H, s, Aromatic CH), 8.67–8.70 (2H, m, Aromatic CH), 12.28 (1H, s, NH). ^13^C‐NMR (100 MHz, DMSO‐d_6_): *δ* = 104.88, 120.39, 121.49, 122.02, 122.50, 123.15, 126.39, 127.35, 128.98, 132.67, 134.98, 138.58, 140.66, 141.93, 146.18, 150.66, 150.88, 163.30. HRMS (*m/z*): [M+H]^+^ calcd for C_20_H_14_N_6_O: 355.1302; found: 355.1297.

4‐(5‐Cyano‐1*H*‐benzo[*d*]imidazol‐2‐yl)‐*N*′‐[(thiophen‐2‐yl)methylene]benzohydrazide (**3g**): Yield: 83%, M.P.= 148.1°C. ^1^H‐NMR (400 MHz, DMSO‐d_6_): δ: 6.15–6.18 (1H, m, Aromatic CH), 6.52 (1H, s, Aromatic CH), 6.94–6.95 (1H, m, Aromatic CH), 7.59–7.62 (2H, m, Aromatic CH), 8.01 (1H, d, *J* = 8.56 Hz, Aromatic CH), 8.11 (2H, d, *J *= 8.52 Hz, Aromatic CH), 8.33–8.35 (3H, m, Aromatic CH), 11.58 (1H, s, NH), 11.72 (1H, s, NH). ^13^C‐NMR (100 MHz, DMSO‐d_6_): *δ *= 104.78, 109.74, 110.21, 114.05, 115.45, 120.44, 123.24, 123.67, 127.30, 127.61, 127.86, 128.14, 128.85, 132.29, 135.86, 141.66, 151.03, 162.54. HRMS (*m/z*): [M+H]^+^ calcd for C_20_H_13_N_5_OS: 372.0914; found: 372.0907.

4‐(5‐Cyano‐1*H*‐benzo[*d*]imidazol‐2‐yl)‐*N*′‐[(pyridine‐3‐yl)methylene]benzohydrazide (**3h**): Yield: 81%, M.P. = 273.4°C. ^1^H‐NMR (400 MHz, DMSO‐d_6_): *δ*: 7.14–7.16 (1H, m, Aromatic CH), 7.49–7.50 (1H, m, Aromatic CH), 7.62–7.69 (3H, m, Aromatic CH), 7.77–7.79 (1H, m, Aromatic CH), 8.10 (3H, d, *J* = 8.36 Hz, Aromatic CH), 8.34 (2H, d, *J* = 8.24 Hz, Aromatic CH), 8.70 (1H, s, Aromatic CH), 11.96 (1H, s, NH), 13.64 (1H, s, NH). ^13^C‐NMR (100 MHz, DMSO‐d_6_): δ= 120.41, 127.36, 128.37, 128.84, 129.60, 131.44, 131.63, 132.32, 132.44, 134.25, 135.37, 138.87, 139.48, 143.75, 150.79, 154.44, 156.27, 162.73. HRMS (*m/z*): [M+H]^+^ calcd for C_21_H_14_N_6_O: 367.1302; found: 367.1297.

4‐(5‐Cyano‐1*H*‐benzo[*d*]imidazol‐2‐yl)‐*N*′‐[(5‐nitrothiophen‐2‐yl)methylene]benzohydrazide (**3i**): Yield: 79%, M.P. = 305.2°C. ^1^H‐NMR (400 MHz, DMSO‐d_6_): *δ*: 7.63–7.64 (1H, m, Aromatic CH), 7.67 (1H, dd, *J*_*1 *_= 1.32 Hz, *J*_*2*_ = 8.32 Hz, Aromatic CH), 7.83–7.84 (1H, m, Aromatic CH), 8.15–8.17 (3H, m, Aromatic CH), 8.23 (1H, br.s., Aromatic CH), 8.39 (2H, d, *J* = 8.36 Hz, Aromatic CH), 8.74 (1H, s, Aromatic CH), 12.38 (1H, s, NH). ^13^C‐NMR (100 MHz, DMSO‐d_6_): *δ* = 111.17, 112.98, 113.82, 120.39, 122.99, 123.80, 127.40, 128.18, 128.98, 130.26, 130.90, 132.85, 134.72, 141.30, 147.02, 151.38, 163.08. HRMS (*m/z*): [M+H]^+^ calcd for C_20_H_12_N_6_O_3_S: 417.0764; found: 417.0762.

4‐(5‐Cyano‐1*H*‐benzo[*d*]imidazol‐2‐yl)‐*N*′‐[(5‐methylthiophen‐2‐yl)methylene]benzohydrazide (**3j**): Yield: 77%, M.P. = 174.2°C. ^1^H‐NMR (400 MHz, DMSO‐d_6_): *δ*: 2.40 (3H, s, CH_3_), 6.75–6.76 (1H, m, Aromatic CH), 7.19–7.20 (1H, m, Aromatic CH), 7.54 (1H, d, *J* = 8.20 Hz, Aromatik CH), 7.68 (1H, br.s., Aromatic CH), 8.01 (2H, d, *J* = 8.44 Hz, Aromatik CH), 8.13 (1H, br.s., Aromatic CH), 8.25 (2H, d, *J *= 8.24 Hz, Aromatik CH), 8.52 (1H, s, Aromatic CH), 11.79 (1H, s, NH). ^13^C‐NMR (100 MHz, DMSO‐d_6_): *δ* = 15.83, 104.79, 120.41, 121.10, 122.24, 124.25, 124.57, 124.98, 126.11, 126.81, 127.33, 128.78, 131.99, 132.37, 135.42, 137.22, 143.60, 143.98, 162.62. HRMS (*m/z*): [M+H]^+^ calcd for C_21_H_15_N_5_OS: 386.1070; found: 386.1065.

### 4.2 Pharmacological/biological assays

#### 4.2.1 Anticancer activity

##### Cell culture

Cell lines from the American Type Culture Collection (ATCC) were used to test the human breast adenocarcinoma cell line (MCF7), rat glial tumor (C6), healthy mouse fibroblast cell line (L929), and colon cancer (HT29). Cells were combined with solutions of 1% penicillin (Sigma Aldrich), 10% fetal bovine serum (FBS; Sigma Aldrich), and 89% DMEM (Dulbecco's modified Eagle's medium; Gibco, Thermo Fisher Scientific). The cells were incubated at 37°C with 95% humidity and 5% CO_2_ to allow the medium to develop.

##### Cell viability assay

All compounds were examined for their cytotoxic effects on the L929, HT29, MCF7, and C6 cell lines using MTT assay. Approximately 1 × 10^4^ cells were seeded in each well. Cells were allowed to adhere for 24 h and then the syntheses were applied at different concentrations. All syntheses were run in three repetitions. Wells with a maximum of 100 µM product were incubated for 24 h. Cisplatin was used as the positive control, and all wells devoid of specimens served as negative controls. To identify metabolically active cells, the wells were incubated for 3 h at 37°C after being treated with MTT solution. Following the MTT interaction, DMSO solution was added to the wells after they had been drained. This solution was used to dissolve the formazan crystals, and a color shift indicated how many live cells were present in each well. With the aid of a microplate, the absorbance values were measured at 540 nm, and the results were displayed as mean ± SD.

#### 4.2.2 Annexin V binding assay

Six‐well plates were seeded with roughly 5 × 10^5^ seeds of each cancer cell, which were left to adhere throughout the night. The next day, compounds **3d** and **3j** were incubated at IC_50_ for another 24 h. Following trypsinization, cells were collected and suspended in phosphate‐buffered saline (PBS) that contained at least 1% FBS. After that, the cells were combined with the Annexin V & Dead Cell reagent according to the manufacturer's instructions. Next, the Muse Cell Analyzer (Millipore) instrument was used to determine the percentage of dead, viable, early, and late apoptotic cells.

##### Cell cycle assay

DNA content (cell cycle) analyses were performed with the MUSE flow cytometry device. When the IC_50_ results were evaluated, compounds **3d** and **3j** caused selective toxicity in the HT29 cancer cell line compared to L929. The same phenomenon was observed with the compound **3d** on breast cancer and glioma cells. Therefore, the main pathways of compounds **3d** and **3j** in cells were determined with the MUSE (cell cycle) kit.

#### 4.2.3 Immunofluorescent microscope analysis

Immunofluorescent microscope analysis was performed as in the previous study.^[^ ^33^ ^]^

#### 4.2.4 RT‐PCR

Determination of Caspase 3, BAX, and BCL‐2 expression profiles in cells applied effective doses of **3d** and/or **3j**:

Total RNA was first isolated from the cells to which effective doses of compounds **3d** and/or **3j** were applied at the 24th hour, by the kit protocols, and then cDNA was synthesized from this total RNA. The expression profiles of Caspase 3, BAX, and BCL‐2 genes from the cDNA samples obtained were analyzed with the RT‐PCR device by the kit protocol. Statistical analysis of the data with the ΔΔCT method was performed using the “RT2 profiler RT‐PCR Array Data Analysis version 3.5” (http://pcrdataanalysissabiosciences.com/pcr/arrayanalysis.php) software.

#### 4.2.5 Carbonic anhydrase IX inhibition assay

CA‐IX level was measured using the DCA900, R&D Systems ELISA Kit. In brief, 100 μL samples were incubated with 50 μL assay buffer for 2 h at room temperature (RT) in a 96‐well plate. The well was then washed four times with 400 µL wash buffer. After 200 µL of a conjugate buffer incubated at room temperature for 2 hours, the plate was washed four times with wash buffer. It was then incubated with 200 µL of substrate buffer for 30 min at room temperature. After washing, 50 µL of the stop solution was used to terminate the reaction. The plate was read at 450 nm using an Epoch™ microplate spectrophotometer (BioTek). The concentration of CA IX protein in each sample was calculated using a standard curve.

#### 4.2.6 Molecular docking

The crystal structure of the CA IX protein (PDB code: 5FL4) was obtained from the Protein Data Bank (www.rcsb.org).^[^ ^34^ ^]^ Since the structure file has nonstandard amino acid numbering, which led to mismatches in identifying residue interactions, the numbering from another CA IX structure (PDB code: 3IAI) was adapted to ensure accurate mapping of residue interactions. All structures were removed using Discovery Studio software except for protein chain A and the metal ion Zn (ZN264).^[^ ^35^ ^]^ Subsequent preparation and docking processes, such as protein and ligand preparation, protonation, grid and search box preparation, force field application, electrostatic calculations, and energy evaluations, were carried out in both re‐docking and docking experiments using the AMDock program, which also uses PDB2PQR, AutoDockZn, and the PyMOL.^[^ ^36^, ^37^, ^38^, ^39^ ^]^ The redocking experiment utilized the “instant coordinates” of the co‐crystallized ligand (5FL4_F_9FK) obtained from the Protein Data Bank as the reference structure. The ligand 9FK is used without additional preprocessing steps to maintain its exact experimental conformation in redocking. The docking protocol was validated after successfully reproducing the co‐crystallized pose with an root mean square deviation of 0.92 Å. The ligands were subjected to energy minimization using the MMFF94s force field for a maximum of 2000 steps, with a convergence criterion set to 1e‐8, using the steepest descent algorithm in Open Babel^[^ ^40^ ^]^ before the docking experiments. The docking experiments used the previously optimized protocol with compounds **3d** and **3j**. The docking simulations were conducted while preserving the metal ion (Zn) in the receptor using AutoDock4Zn and employing the Lamarckian Genetic Algorithm with a maximum of 2,500,000 energy evaluations per run. While preliminary tests with higher evaluations (up to 10,000,000) showed slight improvements in binding affinity, the computational cost was disproportionately high. Hence, the default value was adopted to uphold computational efficiency, with 10 generations and a cluster tolerance set to <2 Å. Coordinates of the search space were determined using the “Center on Residue(s)” option by providing the binding site residues derived from the literature (His 68, Gln 92, His 94, His 96, Glu 106, His 119, Val 121, Arg 129, Val 130, Asp 131, Leu 134, Leu 199, Thr 200, Thr 201; Center:11.08 –29.74 53.98 Size: 29 29 29). All other parameters were maintained as default parameters.

After obtaining the docking poses, we excluded those not meeting the established criteria, such as poor interactions with the active site, insufficient coordination with the Zn (II) ion, or unfavorable binding energies. The remaining poses that met the criteria exhibited average binding energies of approximately –9.0 kcal/mol for 16 and 11 poses of compounds **3d** and **3j**, respectively. Following a visual inspection of the resulting docking poses, we generated interaction fingerprints, representing the frequency of residue interactions within the binding modes. This analysis was performed using the Python tool ProLIF,^[^ ^41^ ^]^ with the “count=True” parameter and a “vicinity cutoff” set to 4.5 Å. Additionally, bond angles and the cyano nitrogen‐Zn (II) distance were calculated based on the first 10 energetically favorable docking poses for each compound (**3d** and **3j**). The observed angles were 117 ± 3.7°, 115 ± 3.4°, and 109 ± 4.7° for **3d**, and 115 ± 4.9°, 116 ± 6.0°, and 110 ± 7.6° for **3j**. The cyano nitrogen‐Zn (II) distance averaged around 2.0 Å. These values were determined using PyMOL^[^ ^39^ ^]^ to assess the geometric relationship between the Zn (II) ion and the cyano nitrogen, which, as suggested in previous structural studies, is a potential ZBG.^[^ ^19^, ^32^, ^42^, ^43^, ^44^ ^]^ While this interaction is well‐documented, its specific role in this context warrants further experimental validation.

## CONFLICTS OF INTEREST STATEMENT

The authors declare no conflicts of interest.

## Supporting information

Supporting information.

Supporting information.

## Data Availability

The data that support the findings of this study are available in the Supporting material of this article.

## References

[1] Q. Lv , J. Zhang , J. Cai , L. Chen , J. Liang , T. Zhang , J. Lin , R. Chen , Z. Zhang , P. Guo , Y. Hong , L. Pan , H. Ji , Chem. Biol. Interact. 2024, 393, 110947. 38479716 10.1016/j.cbi.2024.110947

[2] W. M. Eldehna , E. E. Mohammed , G. H. Al‐Ansary , E. Berrino , M. M. Elbadawi , T. M. Ibrahim , M. Y. Jaballah , S. T. Al‐Rashood , F. A. Binjubair , M. Celik , A. Nocentini , F. A. Elbarbry , F. Sahin , H. A. Abdel‐Aziz , C. T. Supuran , M. Fares , Eur. J. Med. Chem. 2023, 258, 115538. 37321108 10.1016/j.ejmech.2023.115538

[3] I. Koyuncu , E. Temiz , E. M. Güler , M. Durgun , O. Yuksekdag , S. Giovannuzzi , C. T. Supuran , ChemMedChem 2024, 19, e202300680. 38323458 10.1002/cmdc.202300680

[4] S. A. Khan , Z. Shah , S. R. Shah , M. Khan , S. A. Halim , A. Khan , J. Hussain , M. H. Abdellattif , B. Ahmad , A. Al‐Harrasi , Int. J. Biol. Macromol. 2024, 255, 128259. 37984572 10.1016/j.ijbiomac.2023.128259

[5] S. M. Ghouse , K. Sinha , A. Bonardi , G. Pawar , S. Malasala , S. Danaboina , S. Mohammed , V. M. Yaddanapudi , C. T. Supuran , S. Nanduri , Arch. Pharm. 2023, 356(10), 2300316. 10.1002/ardp.202300316 37495909

[6] L. A. Mohammed , M. A. Farhan , S. A. Dadoosh , M. A. Alheety , A. H. Majeed , A. S. Mahmood , Z. H. Mahmoud , SynOpen 2023, 07(04), 652.

[7] İ. Çelik , U. Acar Çevik , K. Küçükoğlu , H. Nadaroglu , H. E. Bostancı , A. Işık , Y. Ozkay , Z. A. Kaplancıklı , Chem. Biol. & Drug Des. 2024, 103(1), e14351. 37697918 10.1111/cbdd.14351

[8] P. Yang , Y. Gu , J. Polym. Sci., Part A: Polym. Chem. 2012, 50(7), 1261.

[9] V. S. Padalkar , B. N. Borse , V. D. Gupta , K. R. Phatangare , V. S. Patil , P. G. Umape , N. Sekar , Arabian J. Chem. 2016, 9, S1125.

[10] R. V. Shingalapur , K. M. Hosamani , R. S. Keri , M. H. Hugar , Eur. J. Med. Chem. 2010, 45(5), 1753. 20122763 10.1016/j.ejmech.2010.01.007

[11] H. P. Vemana , L. Barasa , N. Surubhotla , J. Kong , S. S. C. Ha , J. L. Palaguachi , S. Croft , V. V. Yoganathan , Dukhande , FASEB J. 2019, 33, 646.18.

[12] Y. T. Lee , Y. J. Tan , C. E. Oon , Acta Pharm. Sin. B 2023, 13(2), 478. 36873180 10.1016/j.apsb.2022.09.010 PMC9978992

[13] X. J. Wang , M. Y. Xi , J. H. Fu , F. R. Zhang , G. F. Cheng , D. L. Yin , Q. D. You , Chin. Chem. Lett. 2012, 23(6), 707.

[14] Y. Zhang , J. Xu , Y. Li , H. Yao , X. Wu , Chem. Biol. Drug Des. 2015, 85(5), 541. 25283264 10.1111/cbdd.12442

[15] T. Zhou , T. Guo , Y. Wang , A. Wang , M. Zhang , Chemosphere 2023, 314, 137723. 36592835 10.1016/j.chemosphere.2022.137723

[16] D. H. Lee , M. T. Kim , H. W. Lee , J. H. Han , C. S. Myung , J. Pharm. Investig. 2022, 52(4), 443.

[17] J. Gandasegui , C. Onwuchekwa , A. J. Krolewiecki , S. R. Doyle , R. L. Pullan , W. Enbiale , S. Kepha , H. A. Hatherell , L. van Lieshout , M. Cambra‐Pellejà , V. Escola , J. Muñoz , Lancet Infect. Dis. 2022, 22(11), e341. 35850127 10.1016/S1473-3099(22)00369-3

[18] L. Li , R. Liu , C. Peng , X. Chen , J. Li , Exp. Dermatol. 2022, 31(7), 993. 35538735 10.1111/exd.14602

[19] C. Supuran , Curr. Top. Med. Chem. 2007, 7(9), 825. 17504127 10.2174/156802607780636690

[20] E. Guzel , U. Acar Çevik , A. E. Evren , H. E. Bostancı , U. D. Gul , U. Kayış , Y. Ozkay , Z. A. Kaplancıklı , ACS Omega 2023, 8(4), 4369. 36743066 10.1021/acsomega.2c07755 PMC9893751

[21] H. E. Bostancı , A. T. Bilgiçli , E. Güzel , A. Günsel , C. Hepokur , B. Çimen , M. N. Yarasir , Dalton Trans. 2022, 51(41), 15996. 36200447 10.1039/d2dt01912d

[22] C. Yang , J. Zhang , M. Ding , K. Xu , L. Li , L. Mao , J. Zheng , Clin. Transl. Oncol. 2018, 20(5), 570. 29058263 10.1007/s12094-017-1774-3

[23] P. Familiari , M. Relucenti , P. Lapolla , M. Palmieri , M. Antonelli , L. Cristiano , C. Barbaranelli , M. Catalano , L. D'Angelo , G. Familiari , A. Santoro , A. Frati , P. Bruzzaniti , Biomedicines 1968, 11(7), 2023. 10.3390/biomedicines11071968 PMC10377045 37509607

[24] M. Ergul , Z. Kilic‐Kurt , Y. Aka , O. Kutuk , Z. D. Sahin‐Inan , Toxicol. In Vitro 2024, 95, 105757. 38061602 10.1016/j.tiv.2023.105757

[25] N. O'Donovan , J. Crown , H. Stunell , A. D. Hill , E. McDermott , N. O'Higgins , M. Duffy , J. Clin. Cancer Res. 2003, 9(2), 738. 12576443

[26] V. Alterio , A. Di Fiore , K. D'Ambrosio , C. T. Supuran , G. De Simone , Chem. Rev. 2012, 112(8), 4421. 22607219 10.1021/cr200176r

[27] M. Aggarwal , B. Kondeti , R. McKenna , Bioorg. Med. Chem. 2013, 21(6), 1526. 22985956 10.1016/j.bmc.2012.08.019 PMC3593968

[28] J. Y. Winum , C. T. Supuran , J. Enzyme Inhib. Med. Chem. 2015, 30(2), 321. 24939097 10.3109/14756366.2014.913587

[29] S. Singh , C. Lomelino , M. Mboge , S. Frost , R. McKenna , Molecules 2018, 23(5), 1045. 29710858 10.3390/molecules23051045 PMC6099549

[30] C. T. Supuran , A. Scozzafava , A. Casini , Med. Res. Rev. 2003, 23(2), 146. 12500287 10.1002/med.10025

[31] M. A. Pinard , B. Mahon , R. McKenna , BioMed Res. Int. 2015, 2015, 1. 10.1155/2015/453543 PMC4355338 25811028

[32] Z. Peng , K. M. Merz , L. Banci , Proteins: Struct. Funct. Bioinf. 1993, 17(2), 203. 10.1002/prot.340170209 8265567

[33] U. Acar Çevik , I. Celik , Ş. Görgülü , Z. D. Şahin Inan , H. E. Bostancı , Y. Özkay , Z. A. Kaplacıklı , Drug Dev. Res. 2024, 85(4), e22218. 38825827 10.1002/ddr.22218

[34] H. M. Berman , J. Westbrook, Z. Feng, G. Gilliland, T. N. Bhat, H. Weissig, I. N. Shindyalov, P. E. Bourne, Nucleic Acids Res. 2000, 28(1), 235. 10592235 10.1093/nar/28.1.235 PMC102472

[35] Discovery Studio Visualizer . BIOVIA, Dassault Systèmes; 2024.

[36] M. S. Valdés‐Tresanco , M. E. Valdés‐Tresanco , P. A. Valiente , E. Moreno , Biol. Direct 2020, 15, 12. 32938494 10.1186/s13062-020-00267-2 PMC7493944

[37] T. J. Dolinsky , J. E. Nielsen , J. A. McCammon , N. A. Baker , Nucleic Acids Res. 2004, 32, W665. 15215472 10.1093/nar/gkh381 PMC441519

[38] D. Santos‐Martins , S. Forli , M. J. Ramos , A. J. Olson , J. Chem. Inf. Model. 2014, 54(8), 2371. 24931227 10.1021/ci500209e PMC4144784

[39] The PyMOL Molecular Graphics System (Version 2.1.0), LLC, Schrödinger.

[40] N. M. O'Boyle , M. Banck , C. A. James , C. Morley , T. Vandermeersch , G. R. Hutchison , J. Cheminf. 2011, 3, 1. 10.1186/1758-2946-3-33 PMC3198950 21982300

[41] C. Bouysset , S. J. Fiorucci , Cheminformatics 2021, 13(1), 72. 10.1186/s13321-021-00548-6 PMC8466659 34563256

[42] C. T. Supuran , C. W. Conroy , T. H. Maren , Proteins: Struct. Funct, Genet. 1997, 27(2), 272. 9061790 10.1002/(sici)1097-0134(199702)27:2<272::aid-prot12>3.0.co;2-j

[43] D. West , M. A. Pinard , C. Tu , D. N. Silverman , R. McKenna , Acta Crystallogr. F Struct. Biol. Commun. 2014, 70(10), 1324. 25286933 10.1107/S2053230X14018135 PMC4188073

[44] C. Congiu , V. Onnis , G. Balboni , C. T. Supuran , Bioorg. Med. Chem. Lett. 2014, 24(7), 1776. 24589511 10.1016/j.bmcl.2014.02.030

