# Supplementary material for: New benzimidazole derivatives containing hydrazone group as anticancer agents: Inhibition of carbonic anhydrase IX and molecular docking studies
Source: Arch Pharm (Weinheim). 2025 Mar 24;358(3):e2400930. doi: 10.1002/ardp.202400930 (PMC11931356; doi:10.1002/ardp.202400930)
Supplement: Supplementary file 1 — Supporting information. [file ARDP-358-e2400930-s001.doc]

**New benzimidazole derivatives containing hydrazone group as anticancer agents: Inhibition on carbonic anhydrase IX (CA IX) and molecular docking studies**

Hayrani Eren Bostancı^1^, Mehmet Taha Yıldız^2^, Serkan Kapancık^3^, Zeynep Deniz Şahin Inan^4^, Hacı Ahmet Kılıç^5^, Özen Özensoy Güler^5^, Ulviye Acar Çevik^6*^, Yusuf Özkay^6^, Zafer Asım Kaplancıklı^6,7^

^1^Department of Biochemistry, Faculty of Pharmacy, Cumhuriyet University, Sivas, Turkey

^2^Hamidiye Faculty of Health Sciences, University of Health Sciences, Istanbul, Turkey

^3^Department of Biochemistry, Faculty of Medicine, Cumhuriyet University, Sivas, Turkey

^4^Department of Histology and Embryology, Sivas Cumhuriyet University, Sivas, Turkey

^5^Department of Medical Biology, Faculty of Medicine, Ankara Yildirim Beyazit University, Ankara 06800, Turkey

^6^Department of Pharmaceutical Chemistry, Faculty of Pharmacy, Anadolu University, Eskişehir 26470, Turkey.

^7^The Rectorate of Bilecik Şeyh Edebali University, 11230, Bilecik, Turkey

*Correspondence:

Dr, Ulviye Acar Çevik, Department of Pharmaceutical Chemistry, Faculty of Pharmacy, Anadolu University, 26470, Eskişehir, Türkiye

Email: uacar@anadolu.edu.tr

| **Compound No.** | **InChI** | **Concantration (pg/ml)**  **hCA IX** |
| --- | --- | --- |
| **3a** | InChI=1S/C20H13N5O2/c21-11-13-3-8-17-18(10-13)24-19(23-17)14-4-6-15(7-5-14)20(26)25-22-12-16-2-1-9-27-16/h1-10,12H,(H,23,24)(H,25,26)/b22-12+ | 186,945 |
| **3b** | InChI=1S/C21H15N5O2/c1-13-2-8-17(28-13)12-23-26-21(27)16-6-4-15(5-7-16)20-24-18-9-3-14(11-22)10-19(18)25-20/h2-10,12H,1H3,(H,24,25)(H,26,27)/b23-12+ | 175,225 |
| **3c** | InChI=1S/C20H12N6O4/c21-10-12-1-7-16-17(9-12)24-19(23-16)13-2-4-14(5-3-13)20(27)25-22-11-15-6-8-18(30-15)26(28)29/h1-9,11H,(H,23,24)(H,25,27)/b22-11+ | 150,225 |
| **3d** | InChI=1S/C21H15N5OS/c1-13-8-9-28-19(13)12-23-26-21(27)16-5-3-15(4-6-16)20-24-17-7-2-14(11-22)10-18(17)25-20/h2-10,12H,1H3,(H,24,25)(H,26,27)/b23-12+ | **83,975** |
| **3e** | InChI=1S/C21H14N6O/c22-12-15-1-6-18-19(11-15)26-20(25-18)16-2-4-17(5-3-16)21(28)27-24-13-14-7-9-23-10-8-14/h1-11,13H,(H,25,26)(H,27,28)/b24-13+ | 120,225 |
| **3f** | InChI=1S/C20H14N6O/c21-11-13-3-8-17-18(10-13)25-19(24-17)14-4-6-15(7-5-14)20(27)26-23-12-16-2-1-9-22-16/h1-10,12,22H,(H,24,25)(H,26,27)/b23-12+ | 93,975 |
| **3g** | InChI=1S/C20H13N5OS/c21-11-13-3-8-17-18(10-13)24-19(23-17)14-4-6-15(7-5-14)20(26)25-22-12-16-2-1-9-27-16/h1-10,12H,(H,23,24)(H,25,26)/b22-12+ | 95,745 |
| **3h** | InChI=1S/C21H14N6O/c22-11-14-3-8-18-19(10-14)26-20(25-18)16-4-6-17(7-5-16)21(28)27-24-13-15-2-1-9-23-12-15/h1-10,12-13H,(H,25,26)(H,27,28)/b24-13+ | 119,425 |
| **3ı** | InChI=1S/C20H12N6O3S/c21-10-12-1-7-16-17(9-12)24-19(23-16)13-2-4-14(5-3-13)20(27)25-22-11-15-6-8-18(30-15)26(28)29/h1-9,11H,(H,23,24)(H,25,27)/b22-11+ | 92,225 |
| **3j** | InChI=1S/C21H15N5OS/c1-13-2-8-17(28-13)12-23-26-21(27)16-6-4-15(5-7-16)20-24-18-9-3-14(11-22)10-19(18)25-20/h2-10,12H,1H3,(H,24,25)(H,26,27)/b23-12+ | **82,745** |

^a^ Brief description of screening procedure incl. reference.
