# Supplementary material for: New benzimidazole derivatives containing hydrazone group as anticancer agents: Inhibition of carbonic anhydrase IX and molecular docking studies
Source: Arch Pharm (Weinheim). 2025 Mar 24;358(3):e2400930. doi: 10.1002/ardp.202400930 (PMC11931356; doi:10.1002/ardp.202400930)
Supplement: Supplementary file 2 — Supporting information. [file ARDP-358-e2400930-s002.docx]

**New benzimidazole derivatives containing hydrazone group as anticancer agents: Inhibition on carbonic anhydrase IX (CA IX) and molecular docking studies**

Hayrani Eren Bostancı^1^, Mehmet Taha Yıldız^2^, Serkan Kapancık^3^, Zeynep Deniz Şahin Inan^4^, Hacı Ahmet Kılıç^5^, Özen Özensoy Güler^5^, Ulviye Acar Çevik^6*^, Yusuf Özkay^6^, Zafer Asım Kaplancıklı^6,7^

^1^Department of Biochemistry, Faculty of Pharmacy, Cumhuriyet University, Sivas, Turkey

^2^Hamidiye Faculty of Health Sciences, University of Health Sciences, Istanbul, Turkey

^3^Department of Biochemistry, Faculty of Medicine, Cumhuriyet University, Sivas, Turkey

^4^Department of Histology and Embryology, Sivas Cumhuriyet University, Sivas, Turkey

^5^Department of Medical Biology, Faculty of Medicine, Ankara Yildirim Beyazit University, Ankara 06800, Turkey

^6^Department of Pharmaceutical Chemistry, Faculty of Pharmacy, Anadolu University, Eskişehir 26470, Turkey.

^7^The Rectorate of Bilecik Şeyh Edebali University, 11230, Bilecik, Turkey

* Corresponding Author. E-mail: uacar@anadolu.edu.tr; Tel. +90-222-335-0580/3775

Address: Anadolu University, Faculty of Pharmacy, Department of Pharmaceutical Chemistry, 26470, Eskişehir, Turkey.

**
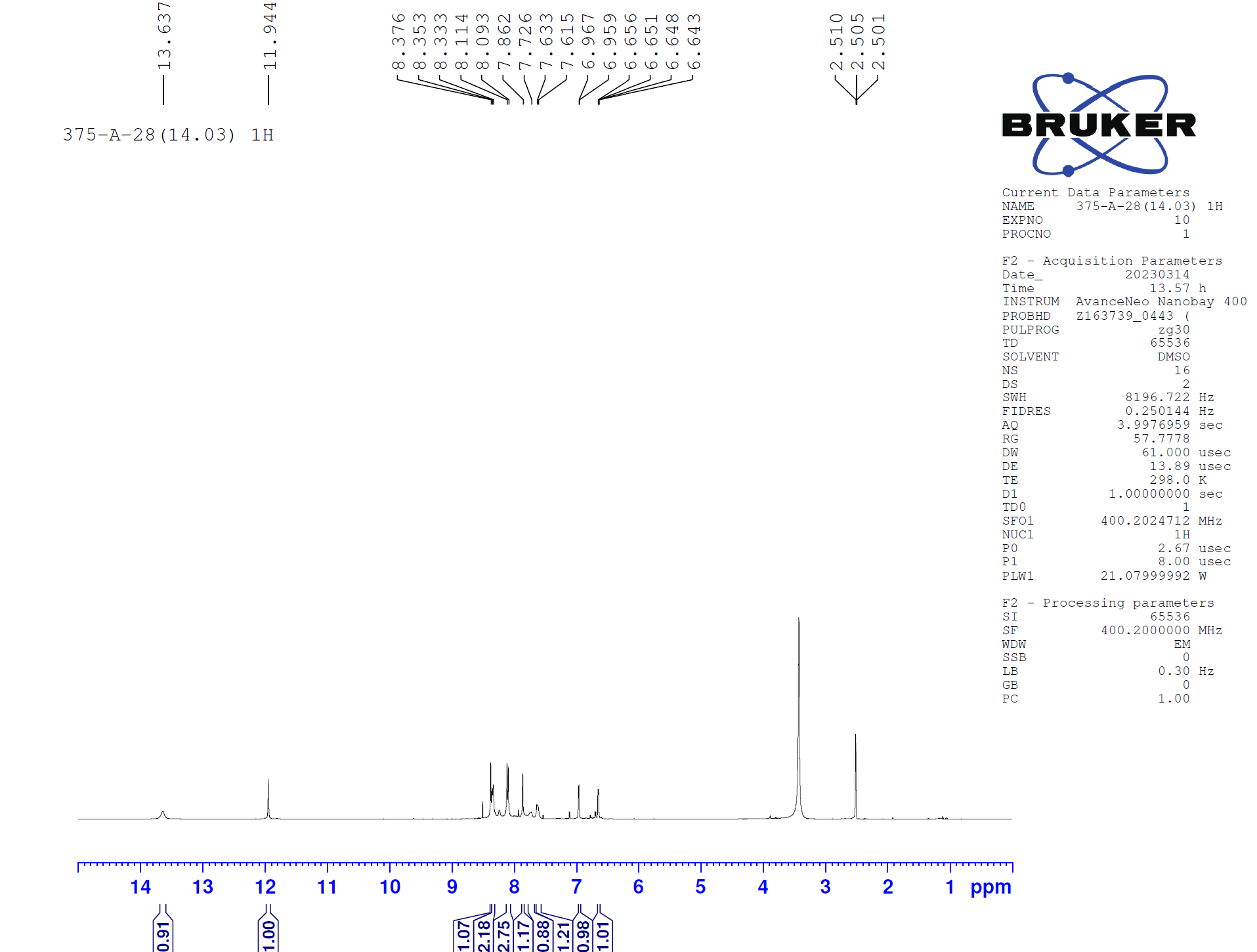
Figure 1.** ^1^H-NMR spectrum of compound **3a**

**
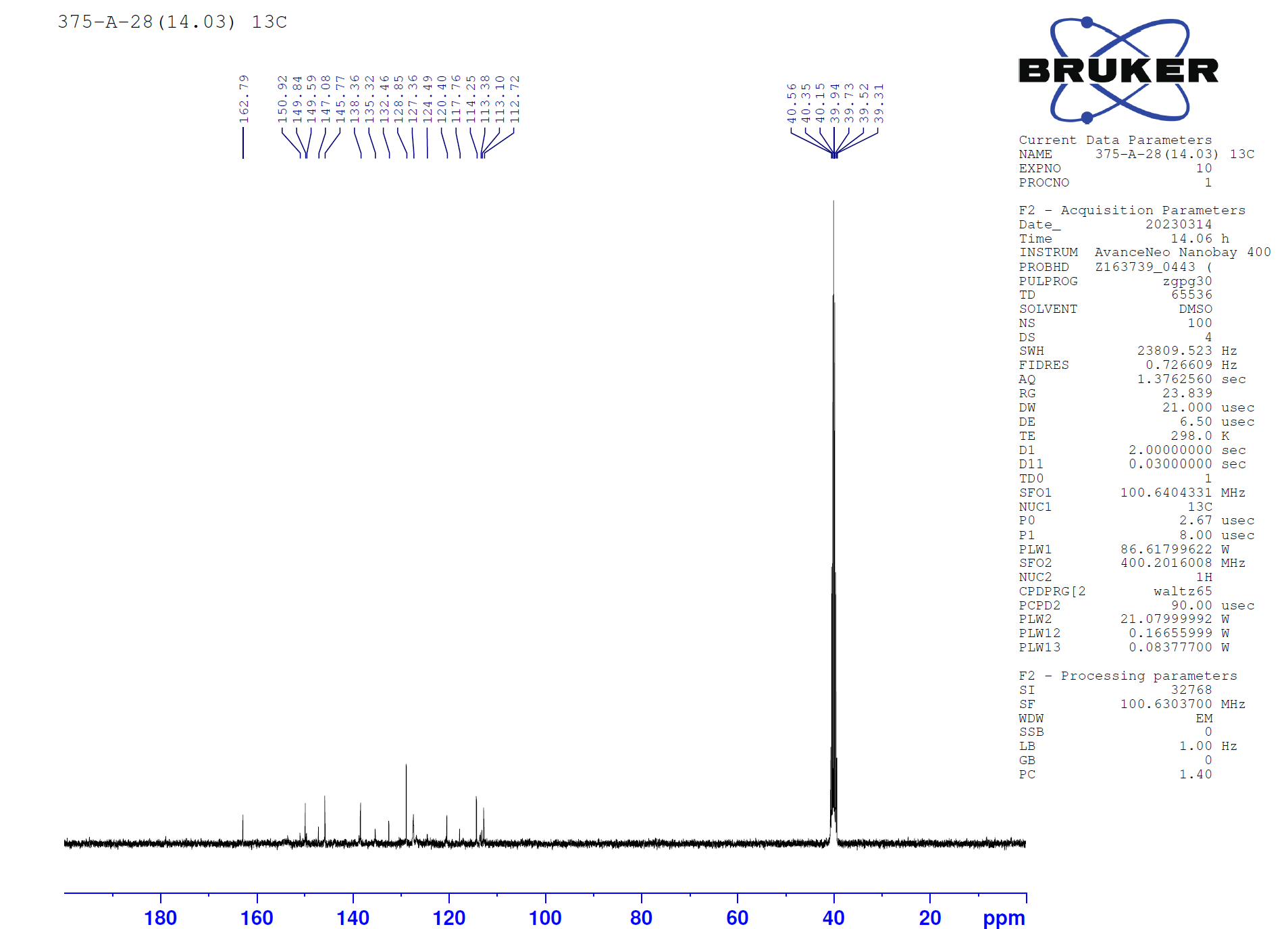
Figure 2.** ^13^C-NMR spectrum of compound **3a**


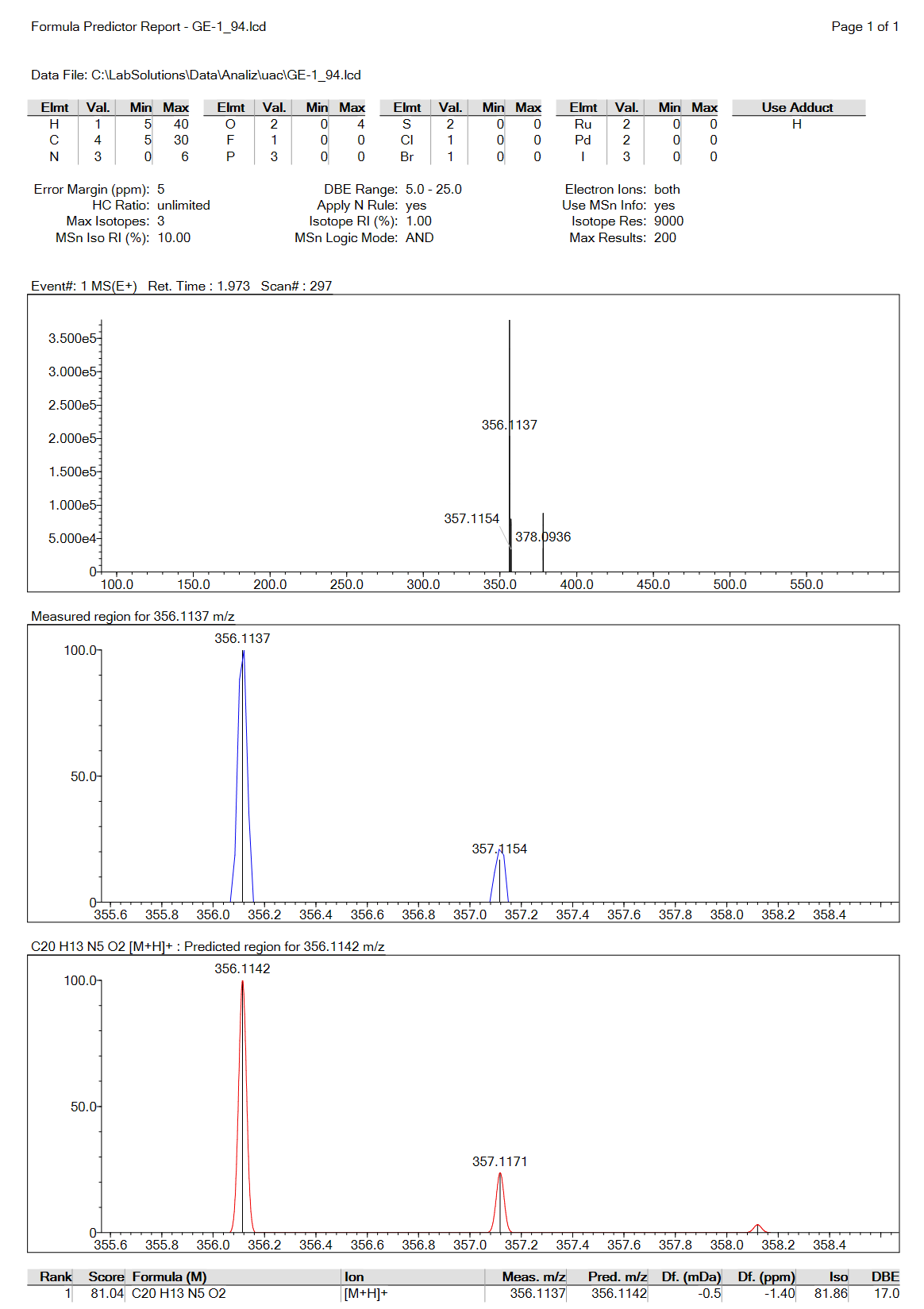


**Figure 3.** Mass spectrum of compound **3a**


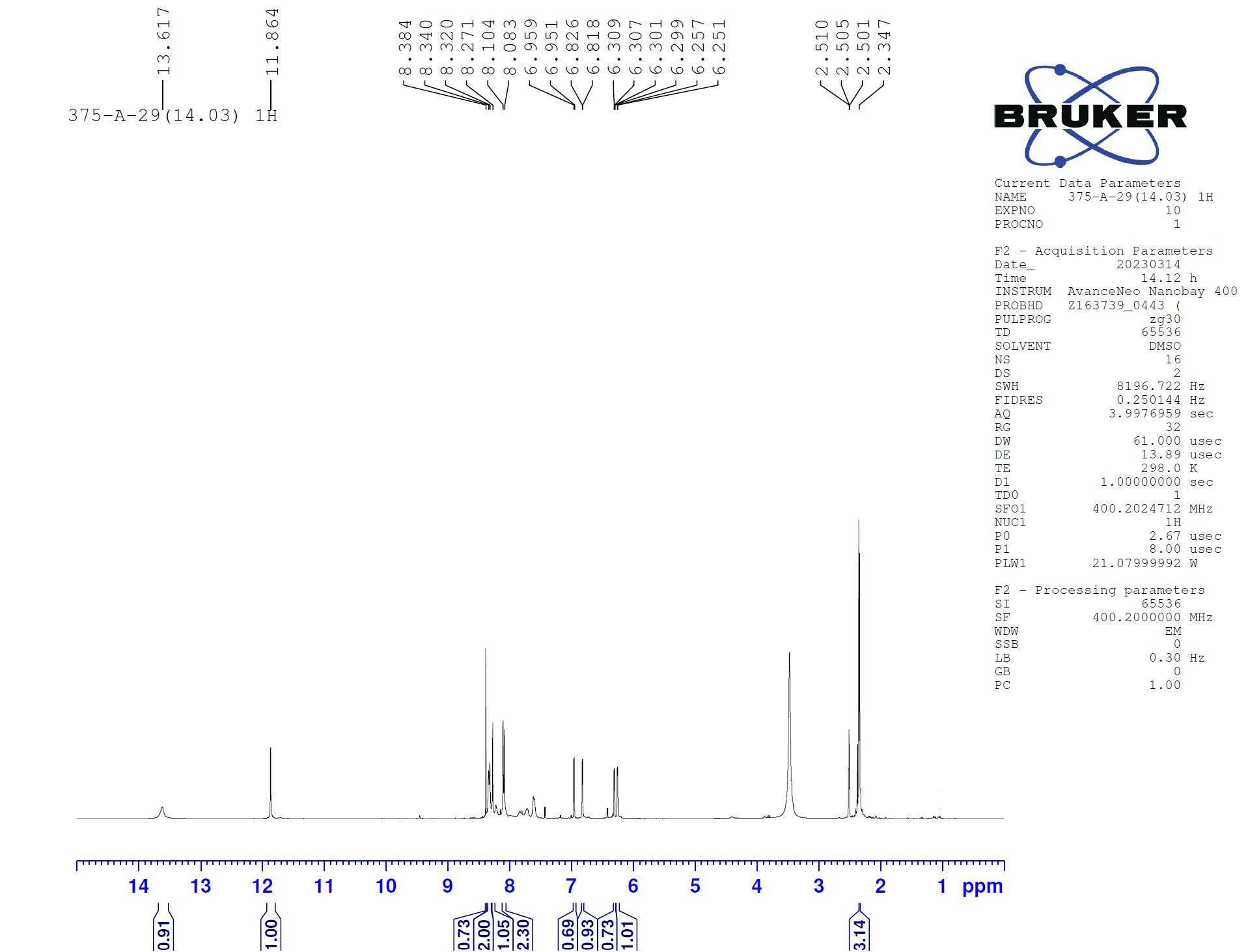


**Figure 4.** ^1^H-NMR spectrum of compound **3b**

**
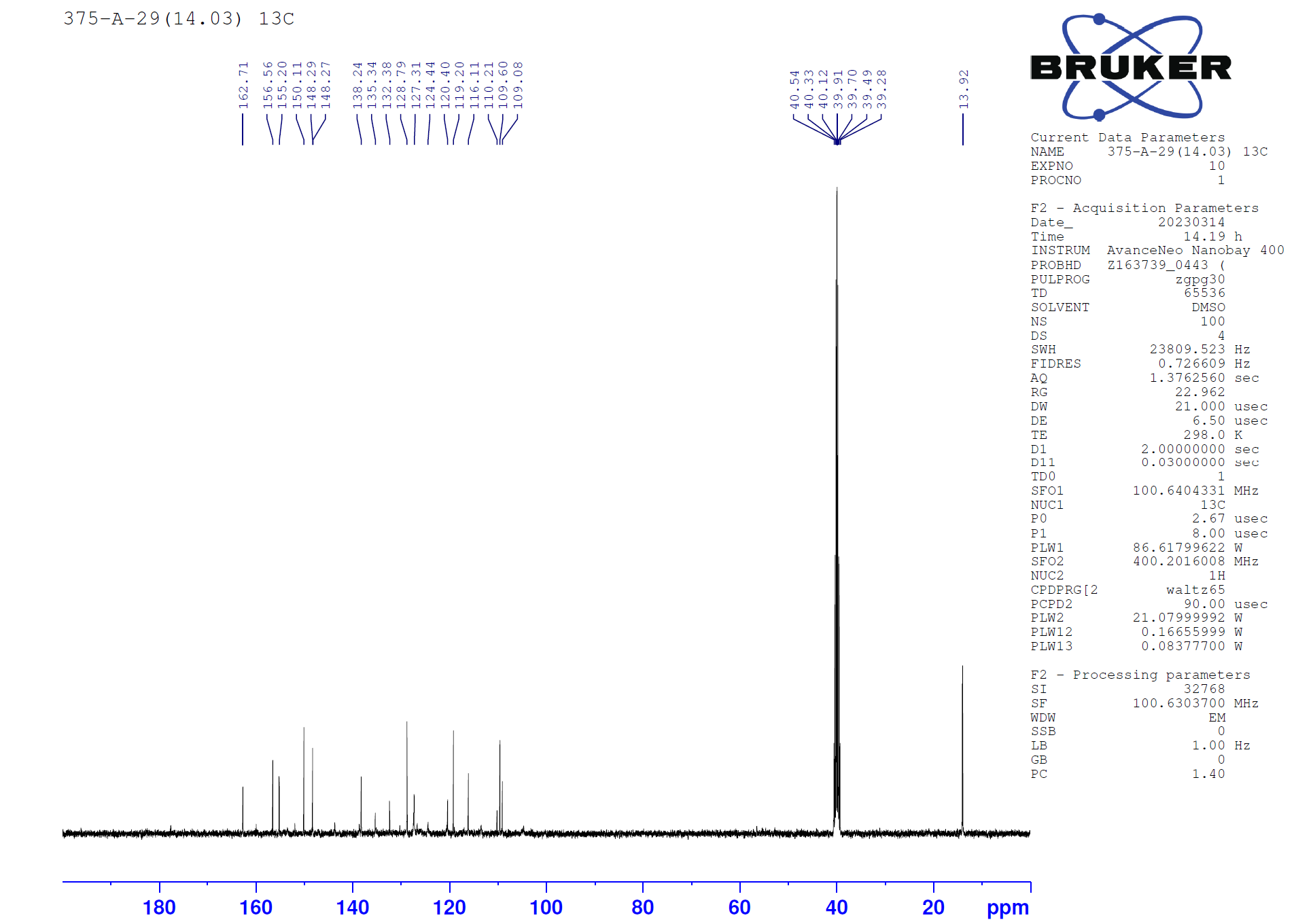
Figure 5.** ^13^C-NMR spectrum of compound **3b**


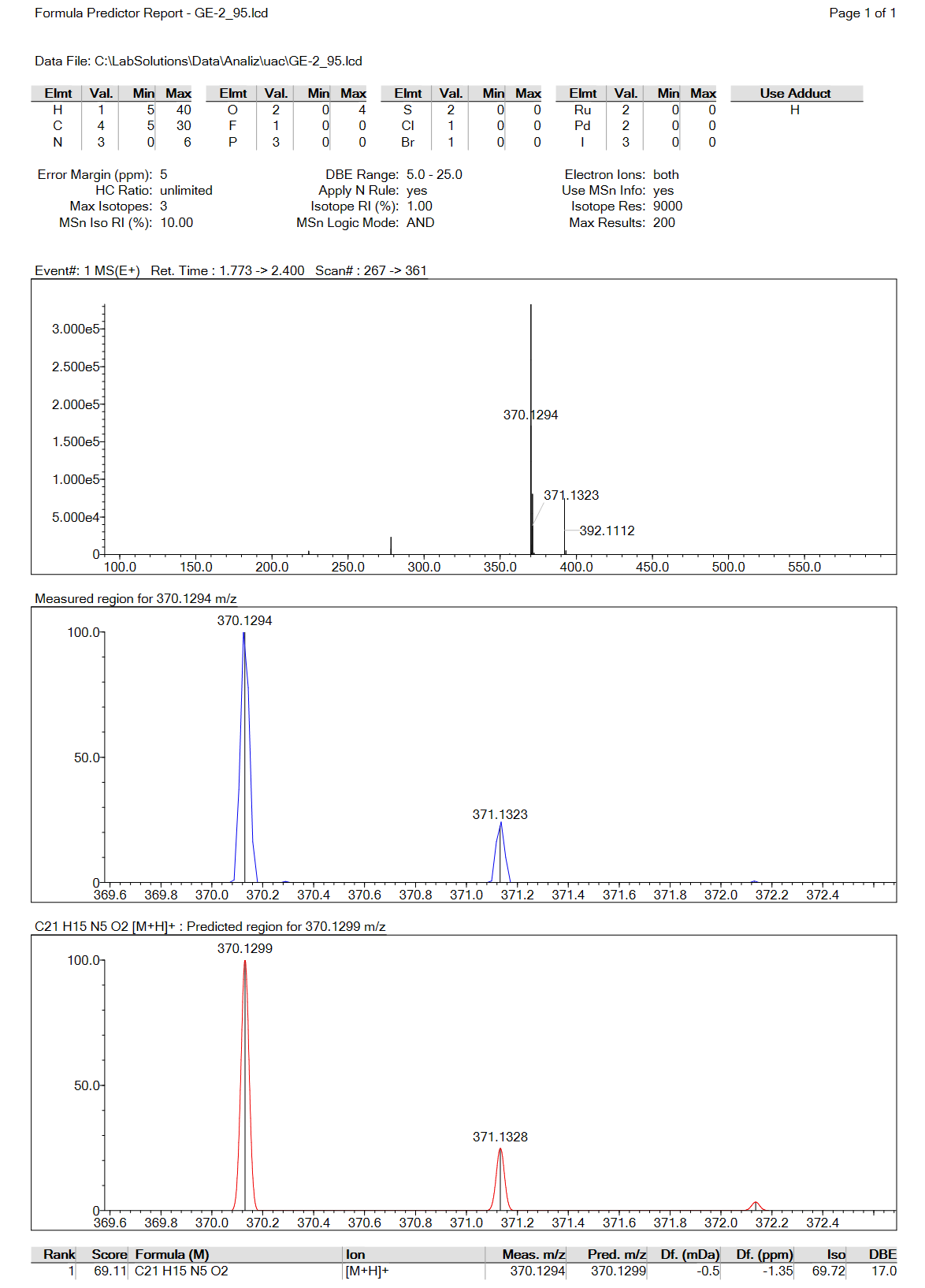


**Figure 6.** Mass spectrum of compound **3b**

**
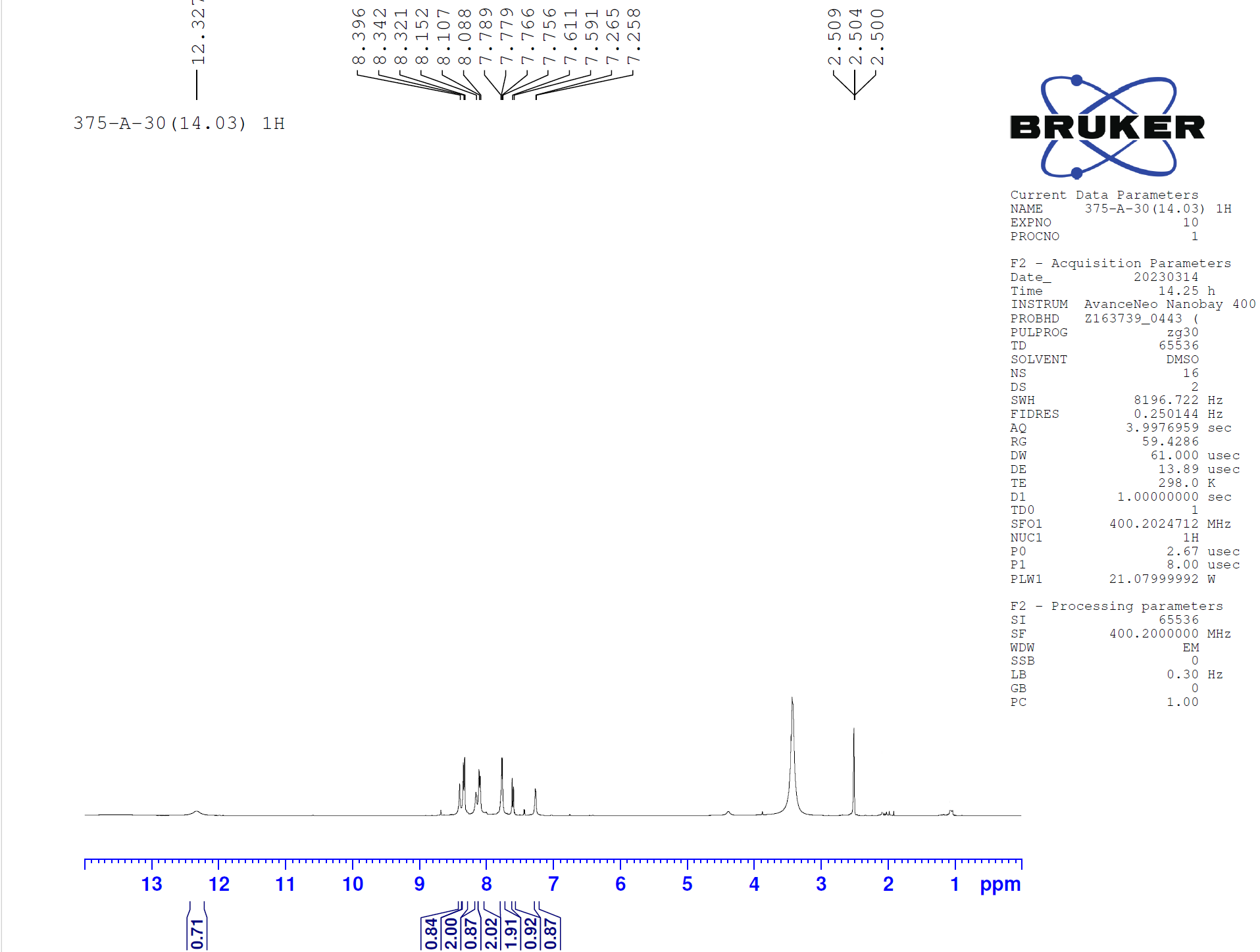
**

**Figure 7.** ^1^H-NMR spectrum of compound **3c**

**
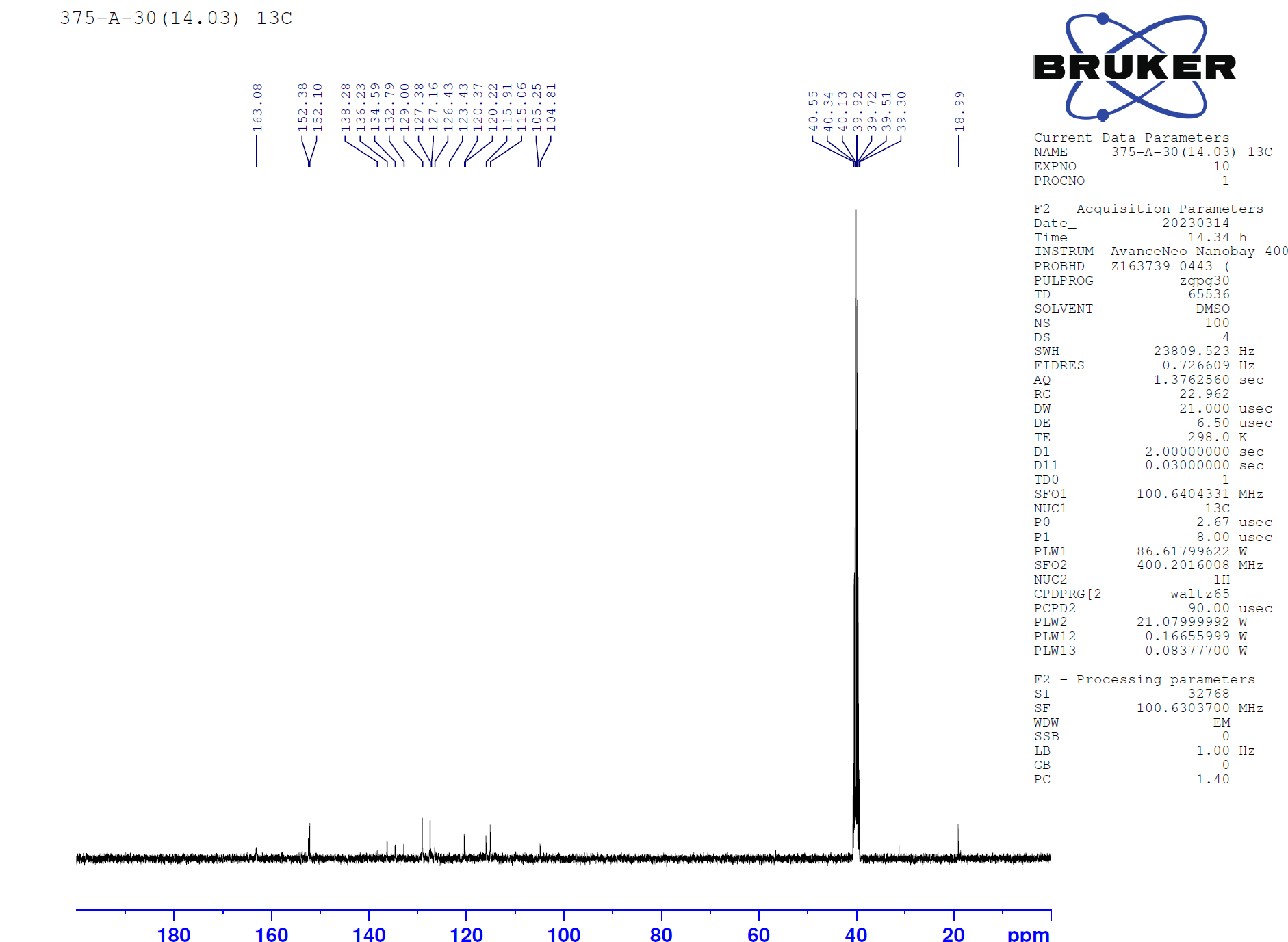
**

**Figure 8.** ^13^C-NMR spectrum of compound **3c**


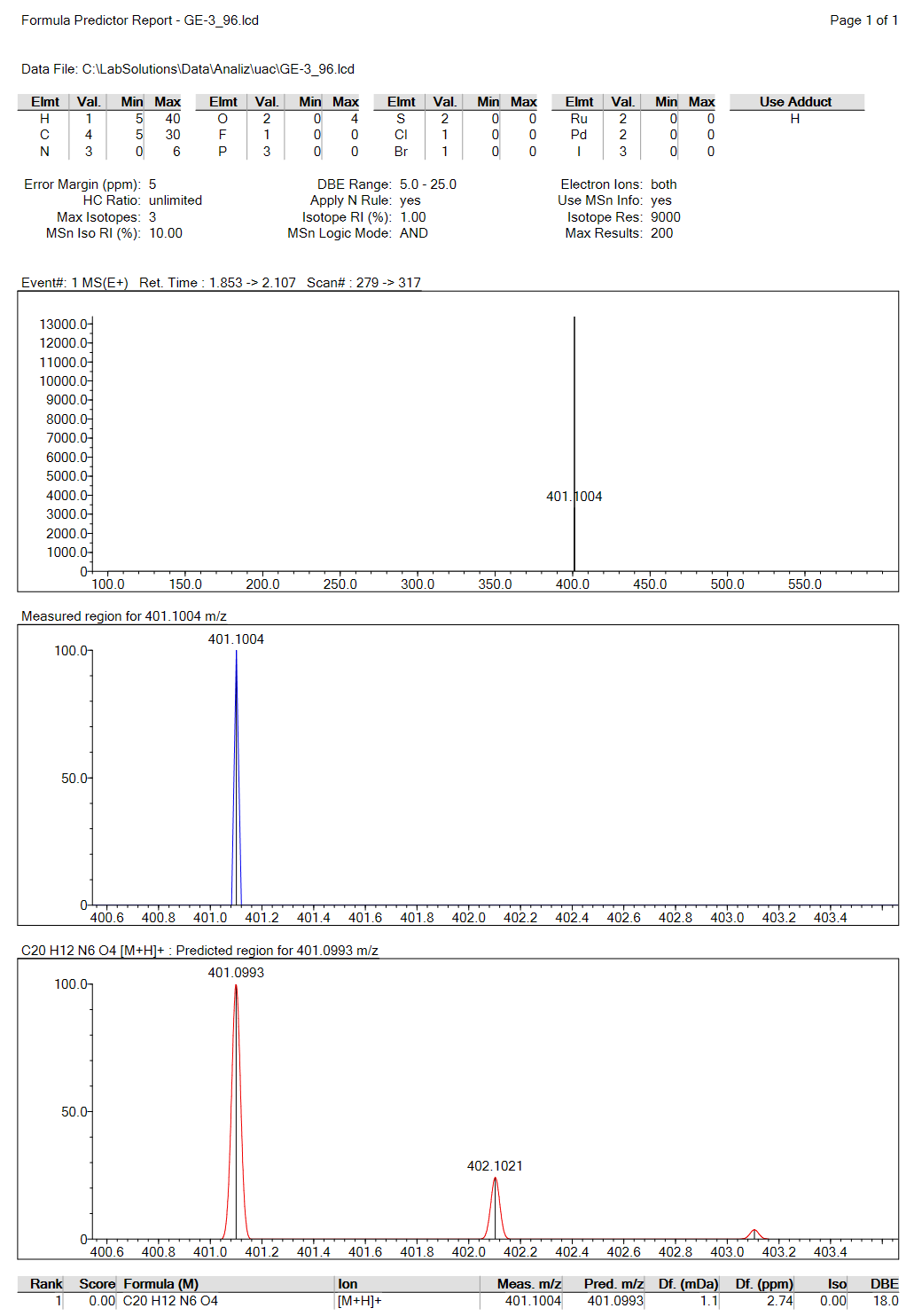


**Figure 9.** Mass spectrum of compound **3c**

**
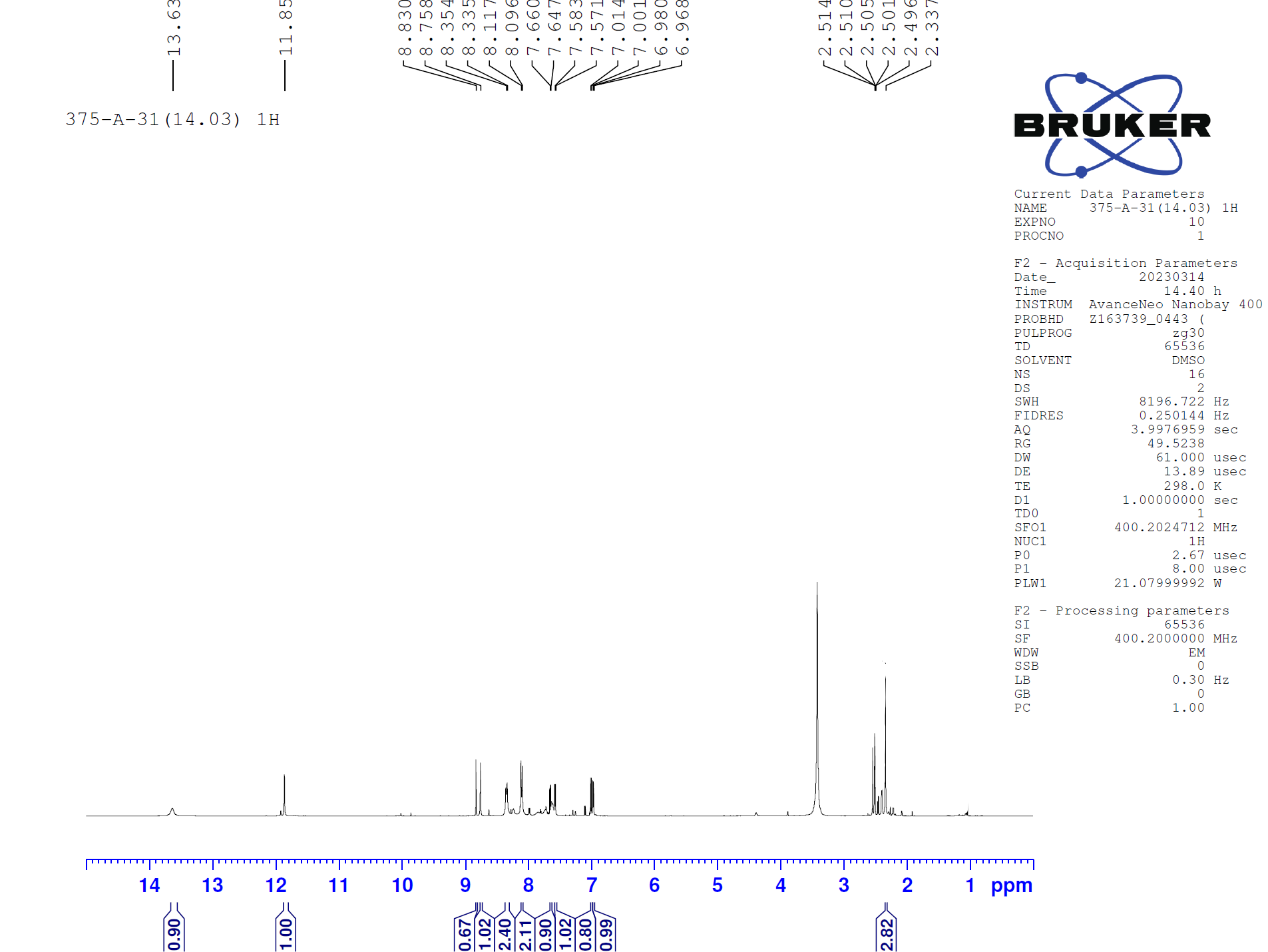
**

**Figure 10.** ^1^H-NMR spectrum of compound **3d**

**
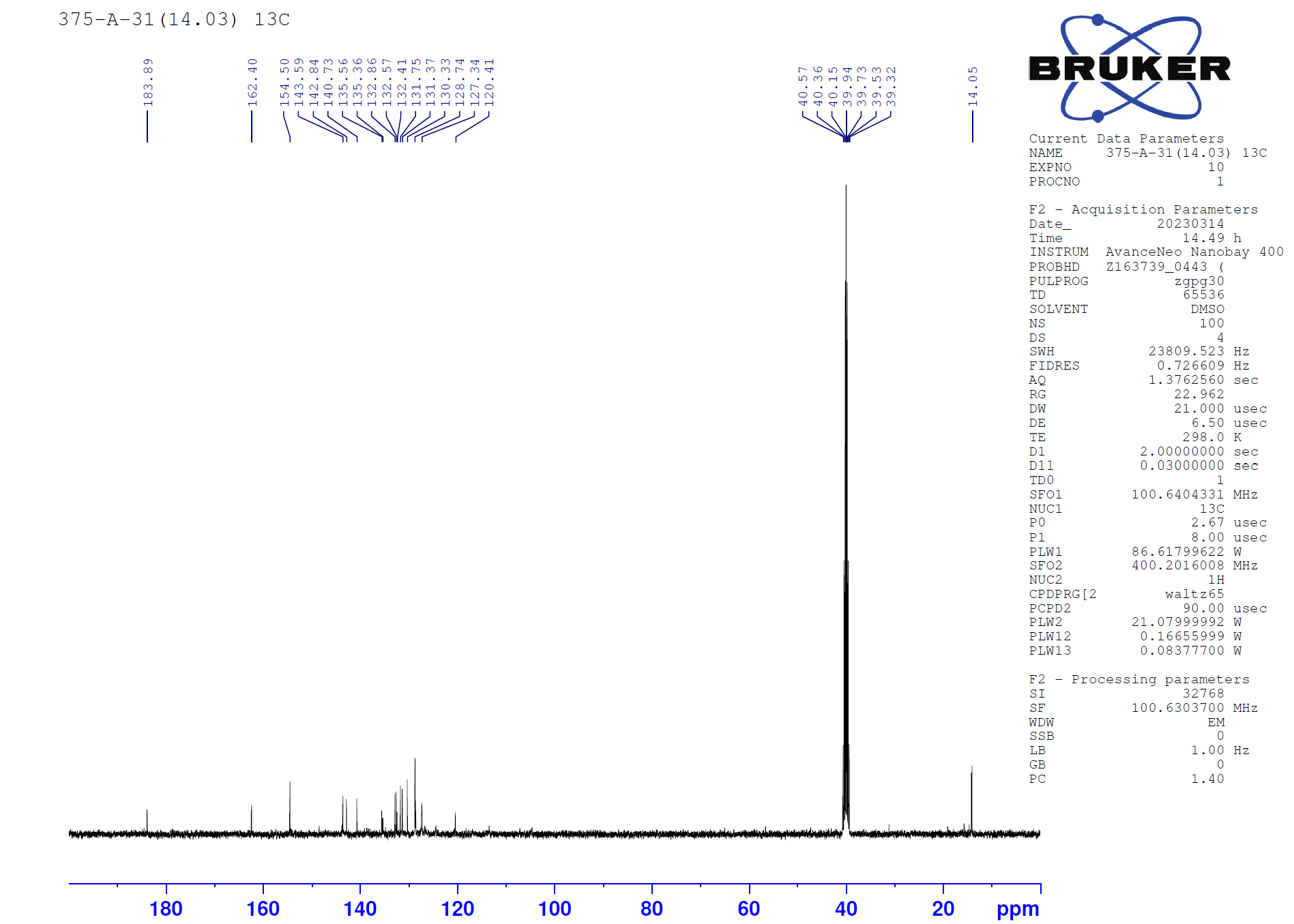
Figure 11.** ^13^C-NMR spectrum of compound **3d**


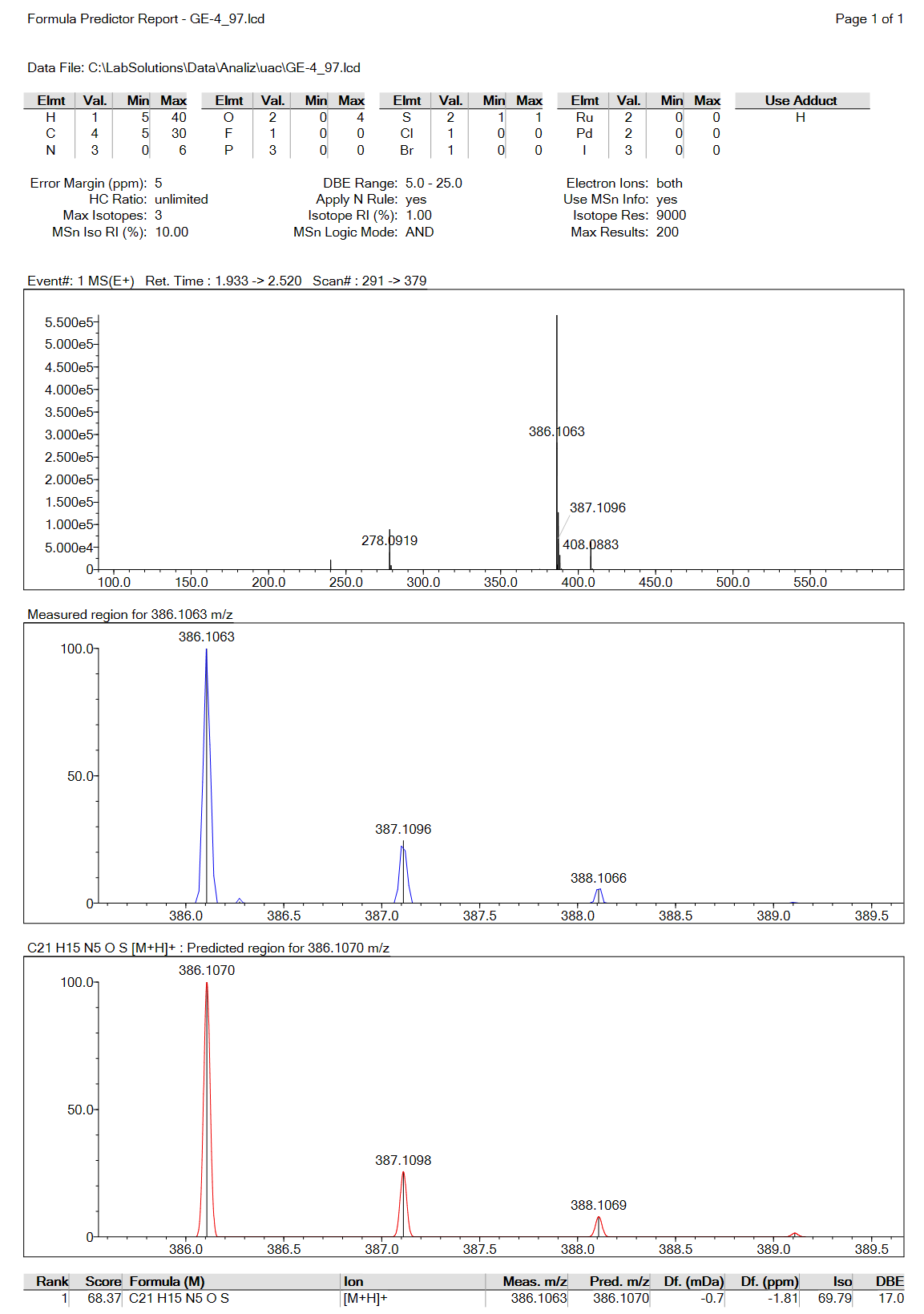


**Figure 12.** Mass spectrum of compound **3d**

**
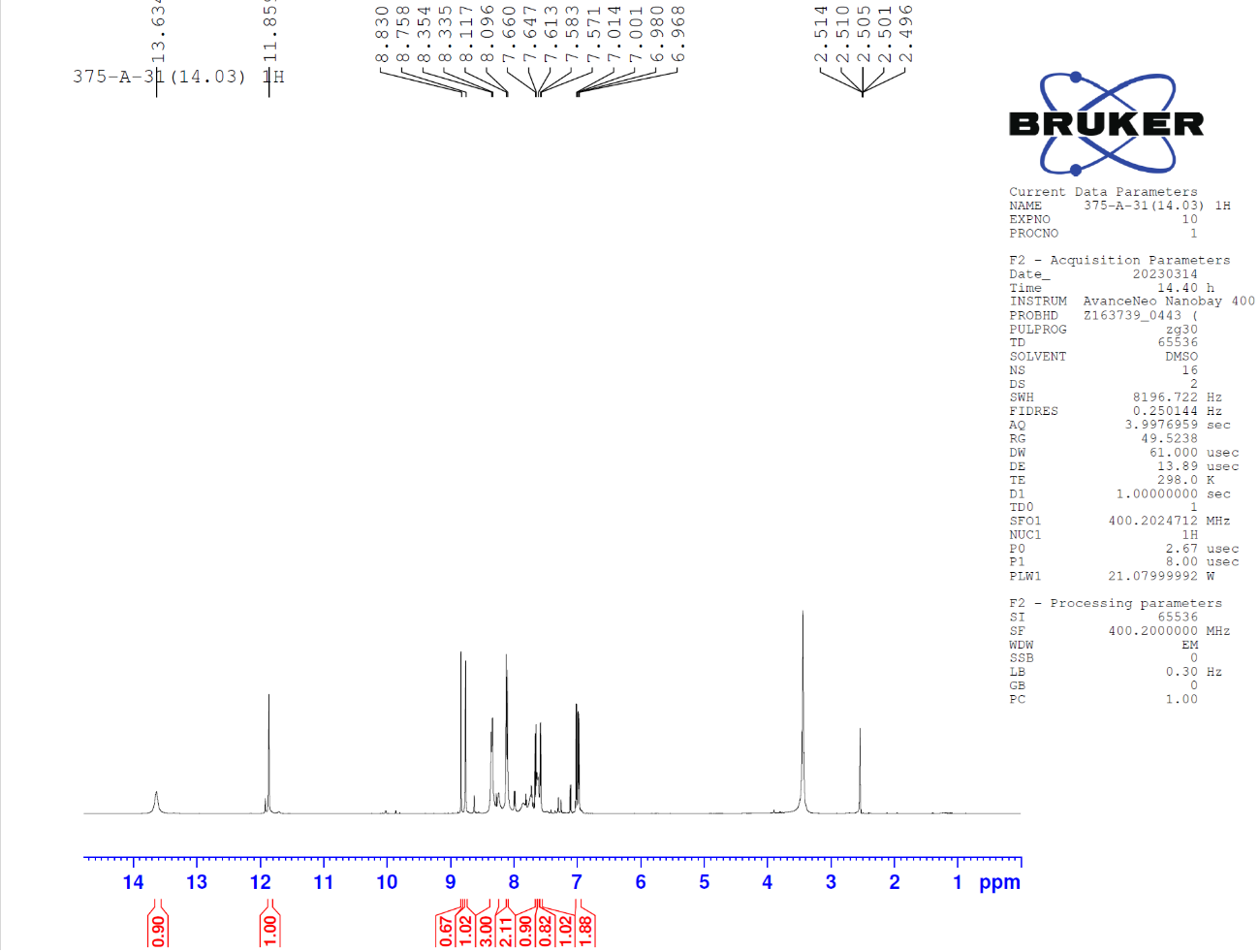
**

**Figure 13.** ^1^H-NMR spectrum of compound **3e**

**
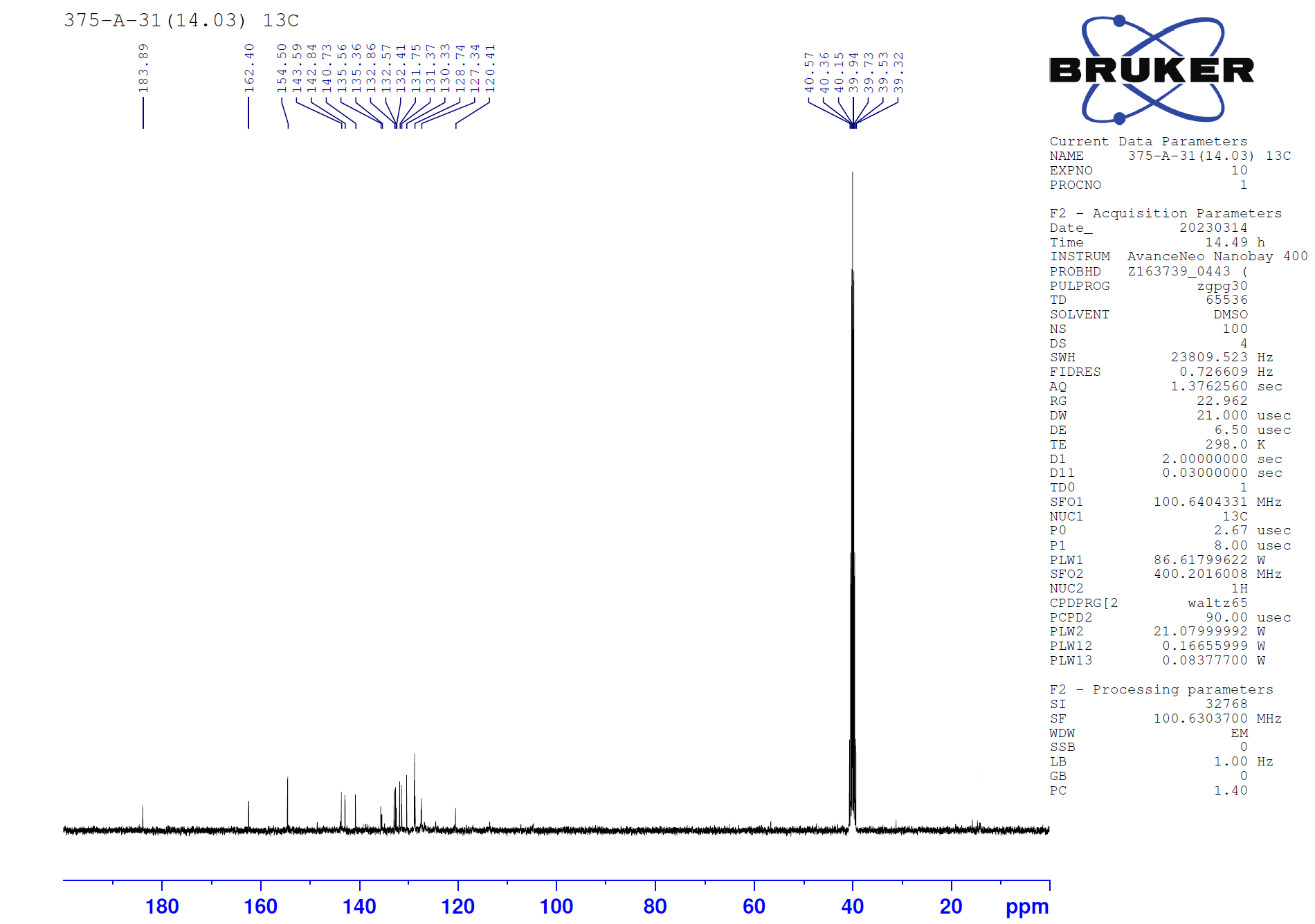
Figure 14.** ^13^C-NMR spectrum of compound **3e**


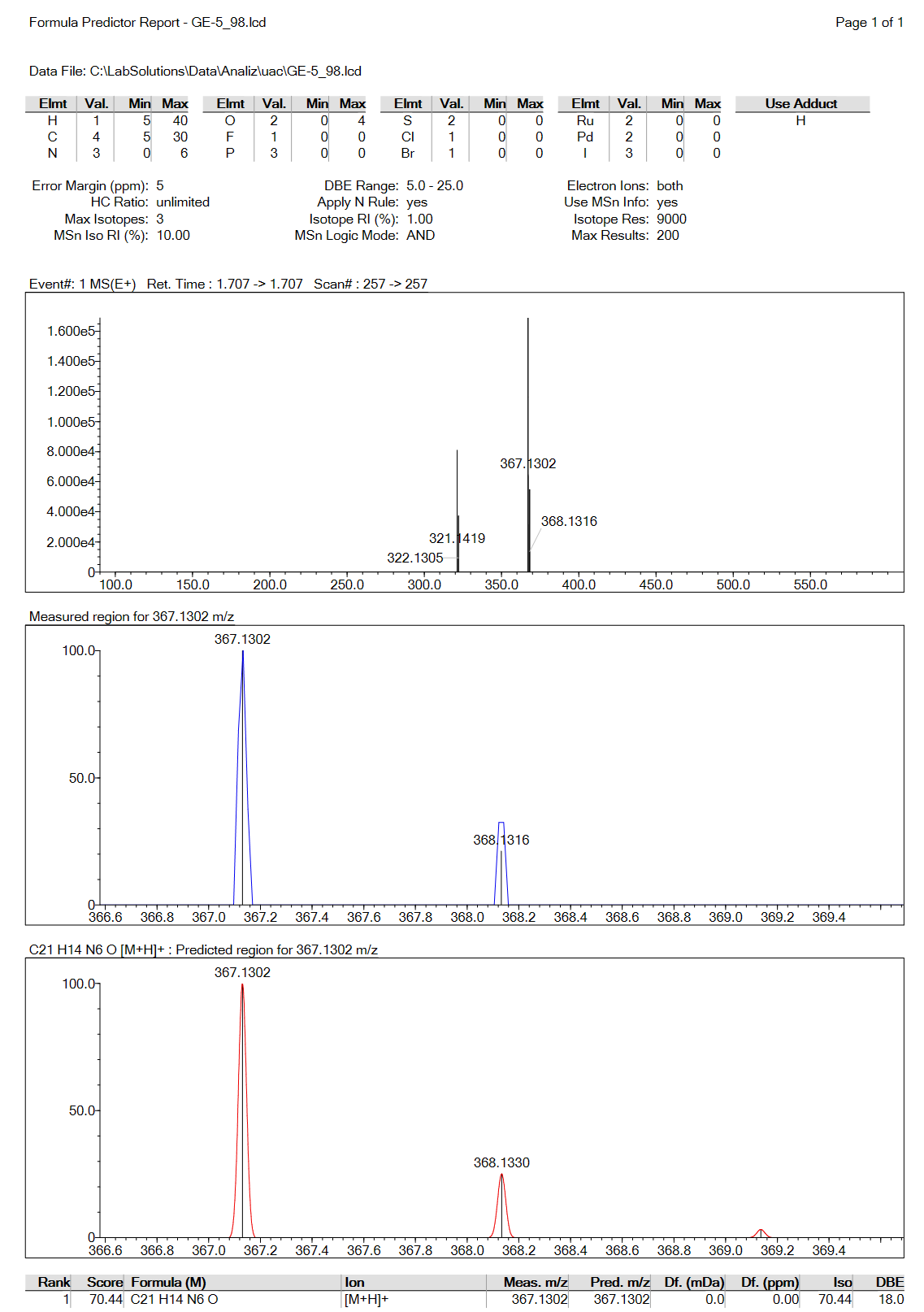


**Figure 15.** Mass spectrum of compound **3e**

**
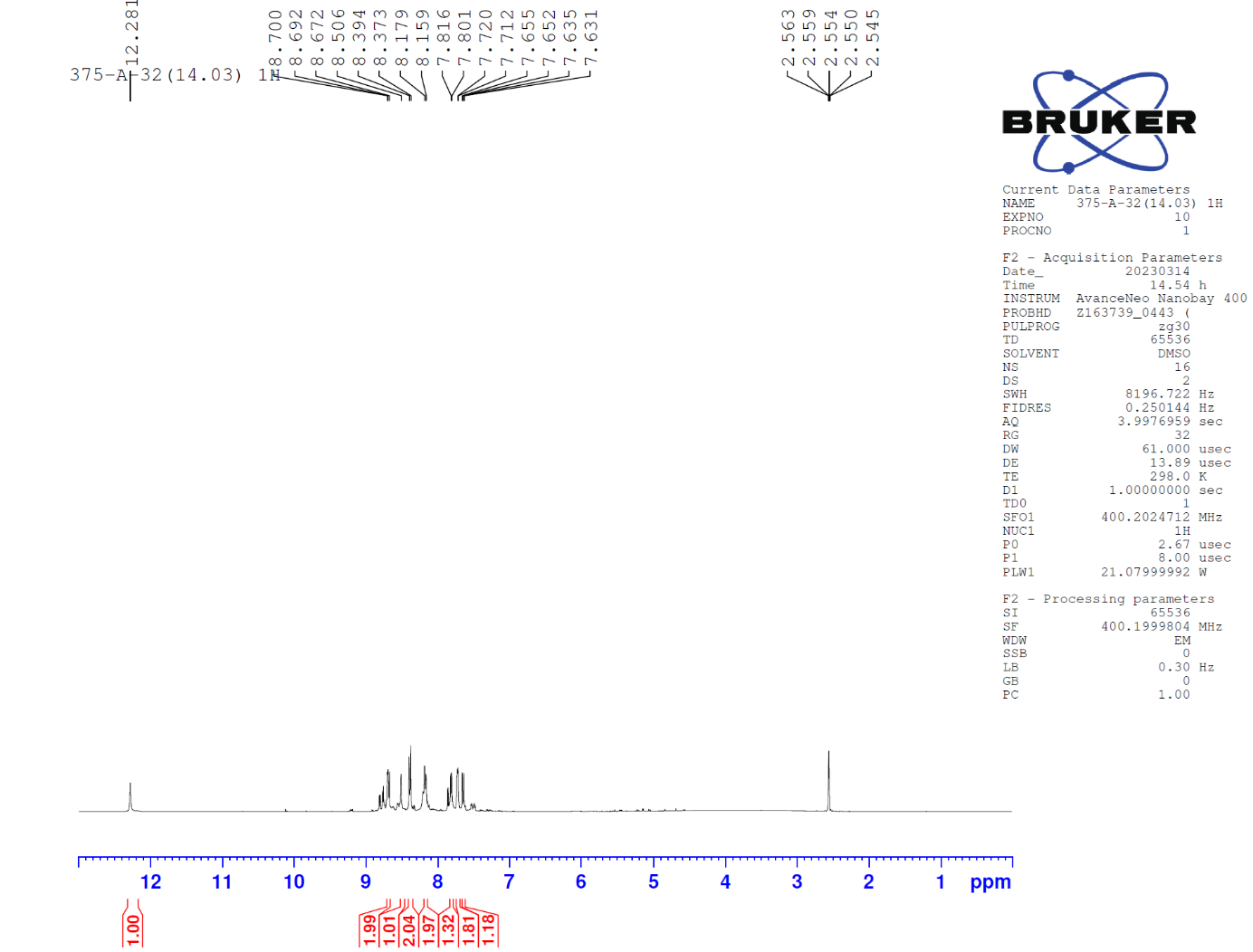
**

**Figure 16.** ^1^H-NMR spectrum of compound **3f**

**
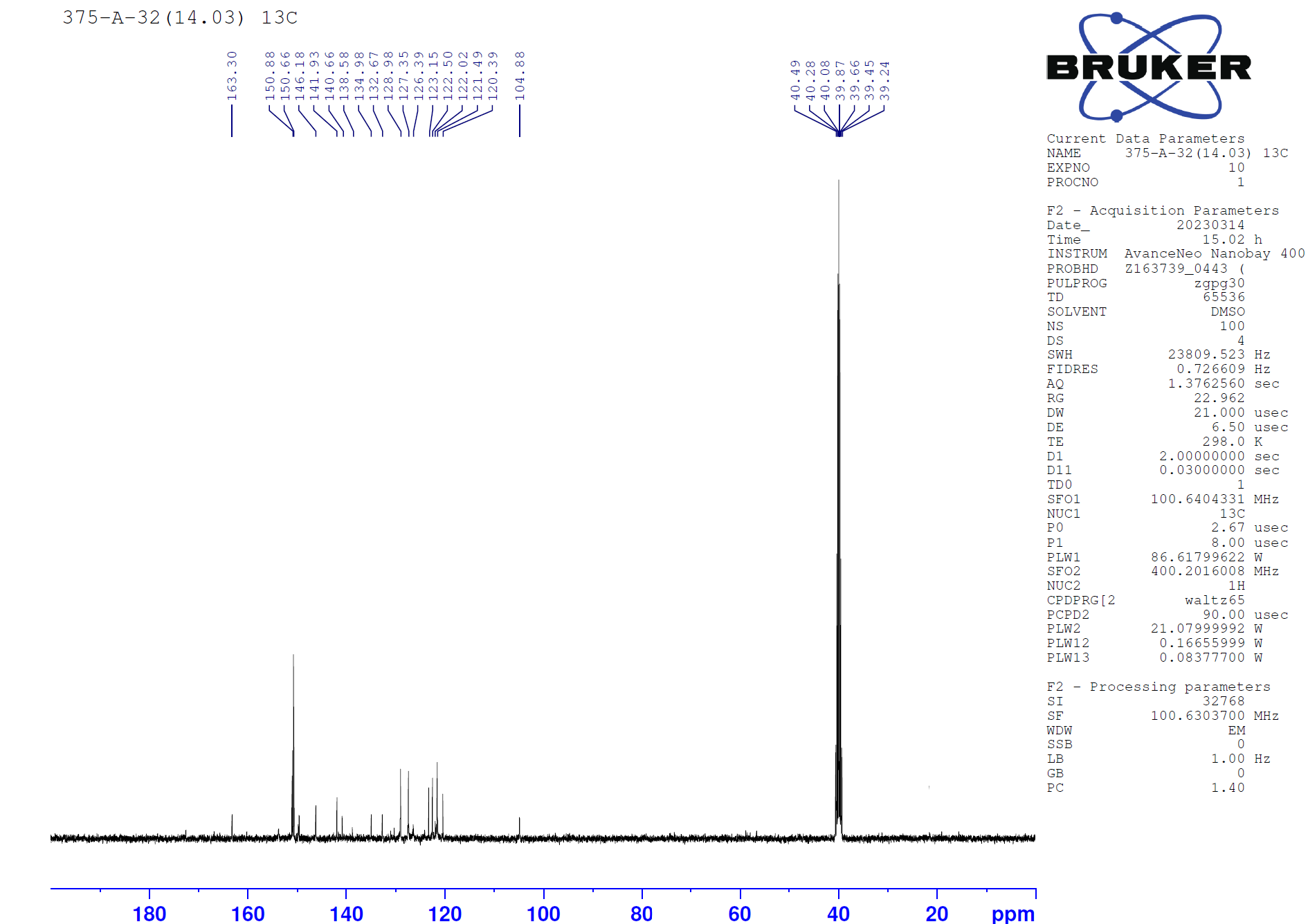
Figure 17.** ^13^C-NMR spectrum of compound **3f**


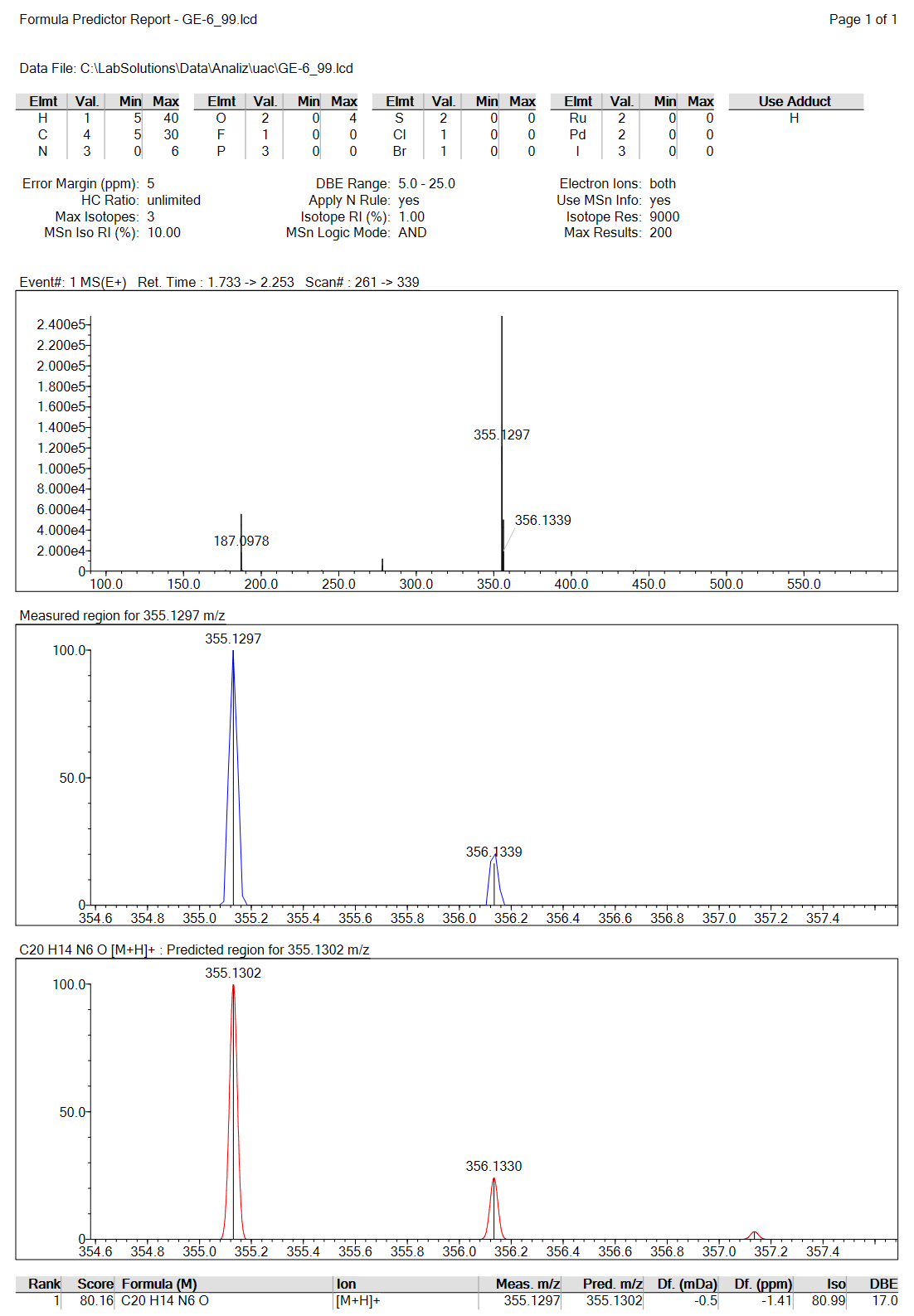


**Figure 18.** Mass spectrum of compound **3f**

**
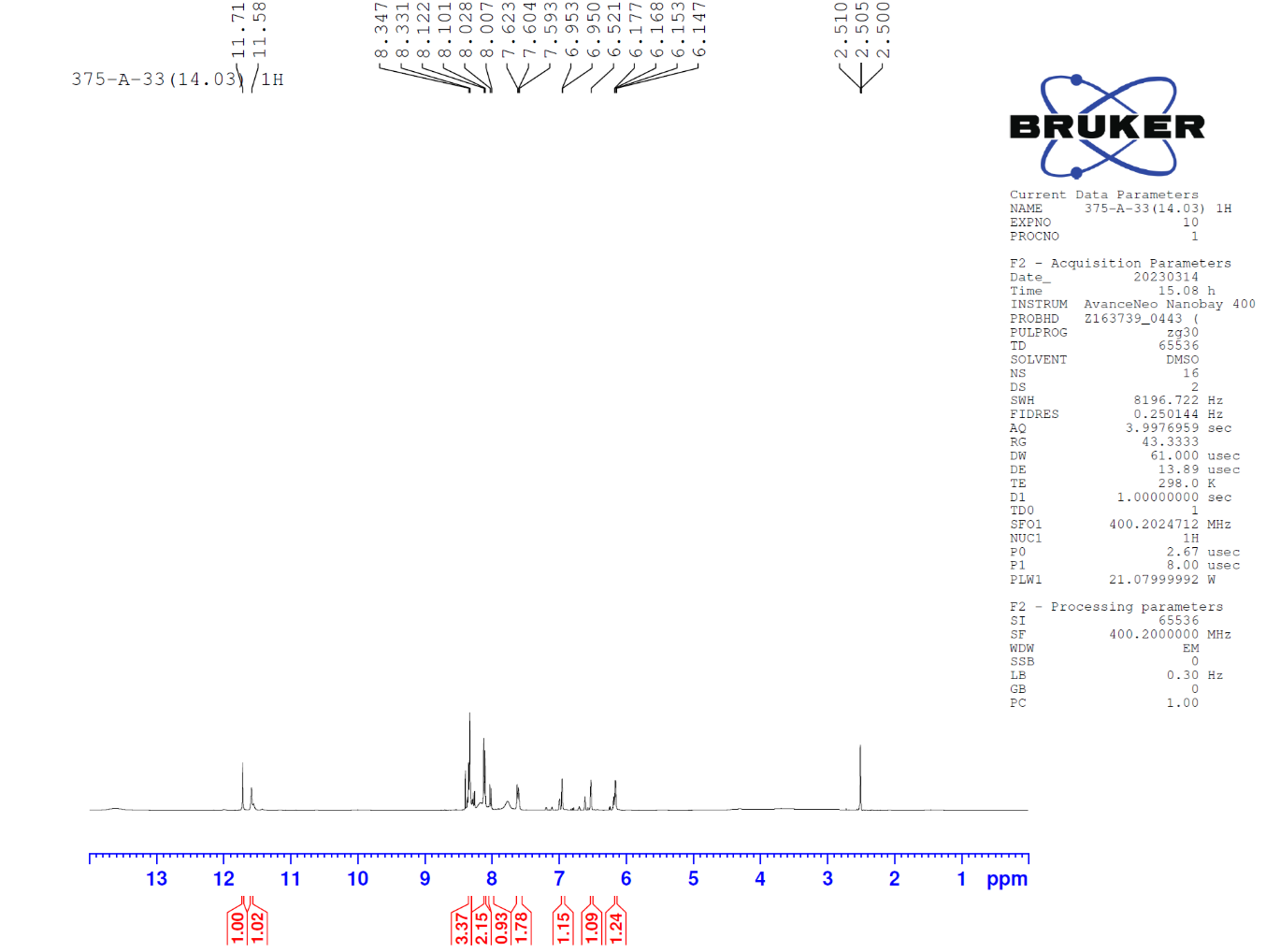
**

**Figure 19.** ^1^H-NMR spectrum of compound **3g**

**
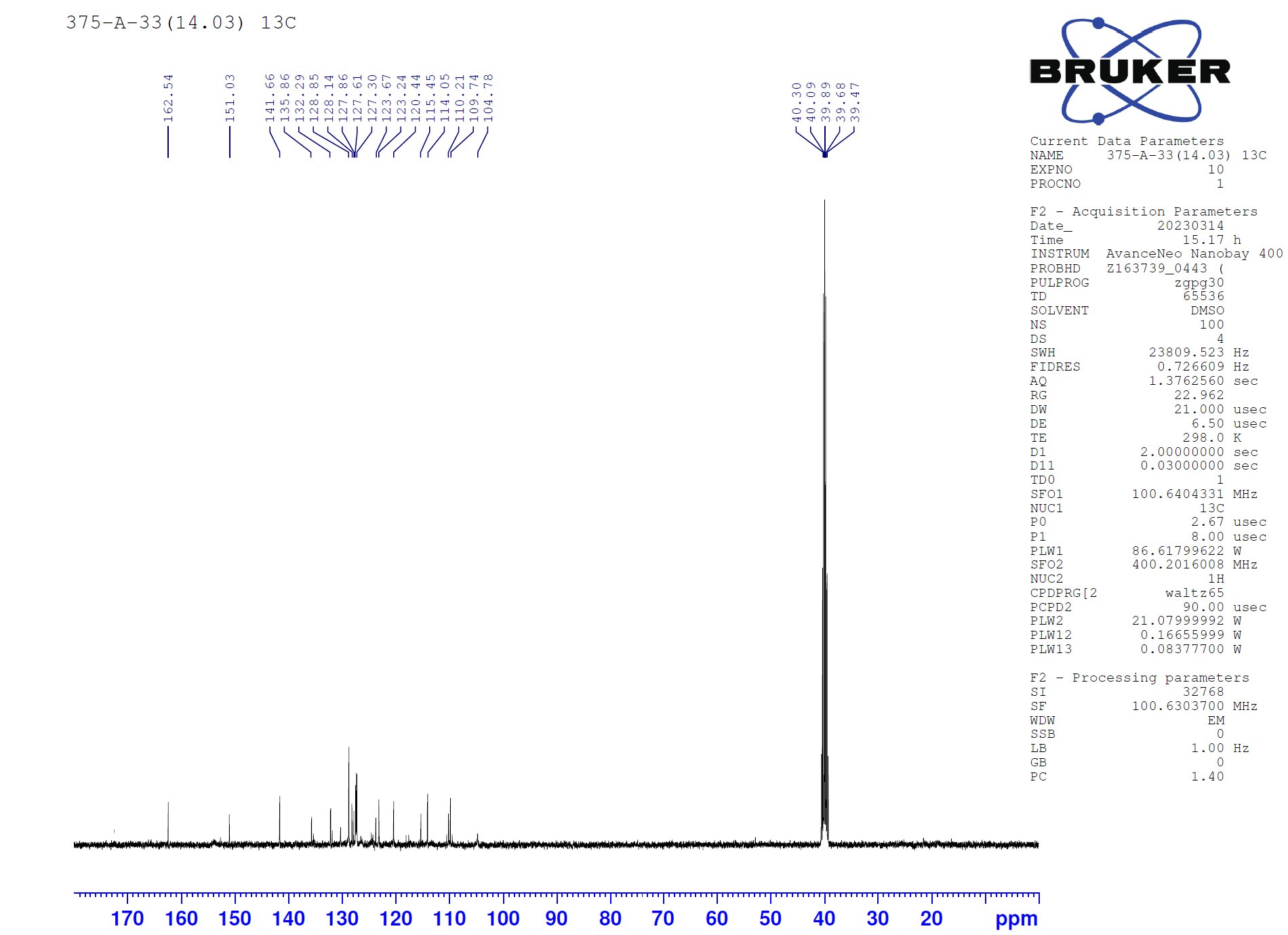
Figure 20.** ^13^C-NMR spectrum of compound **3g**


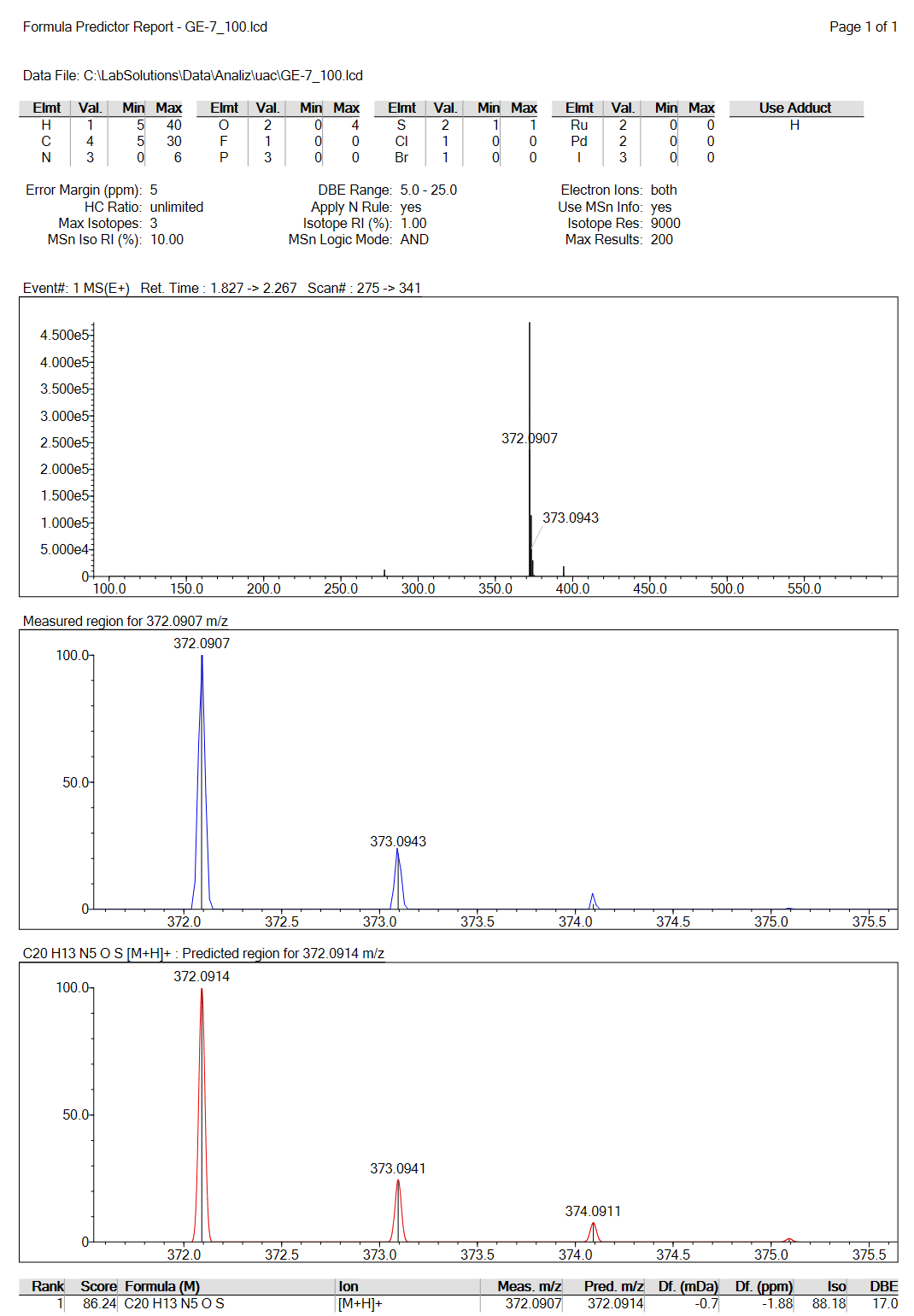


**Figure 21.** Mass spectrum of compound **3g**

**
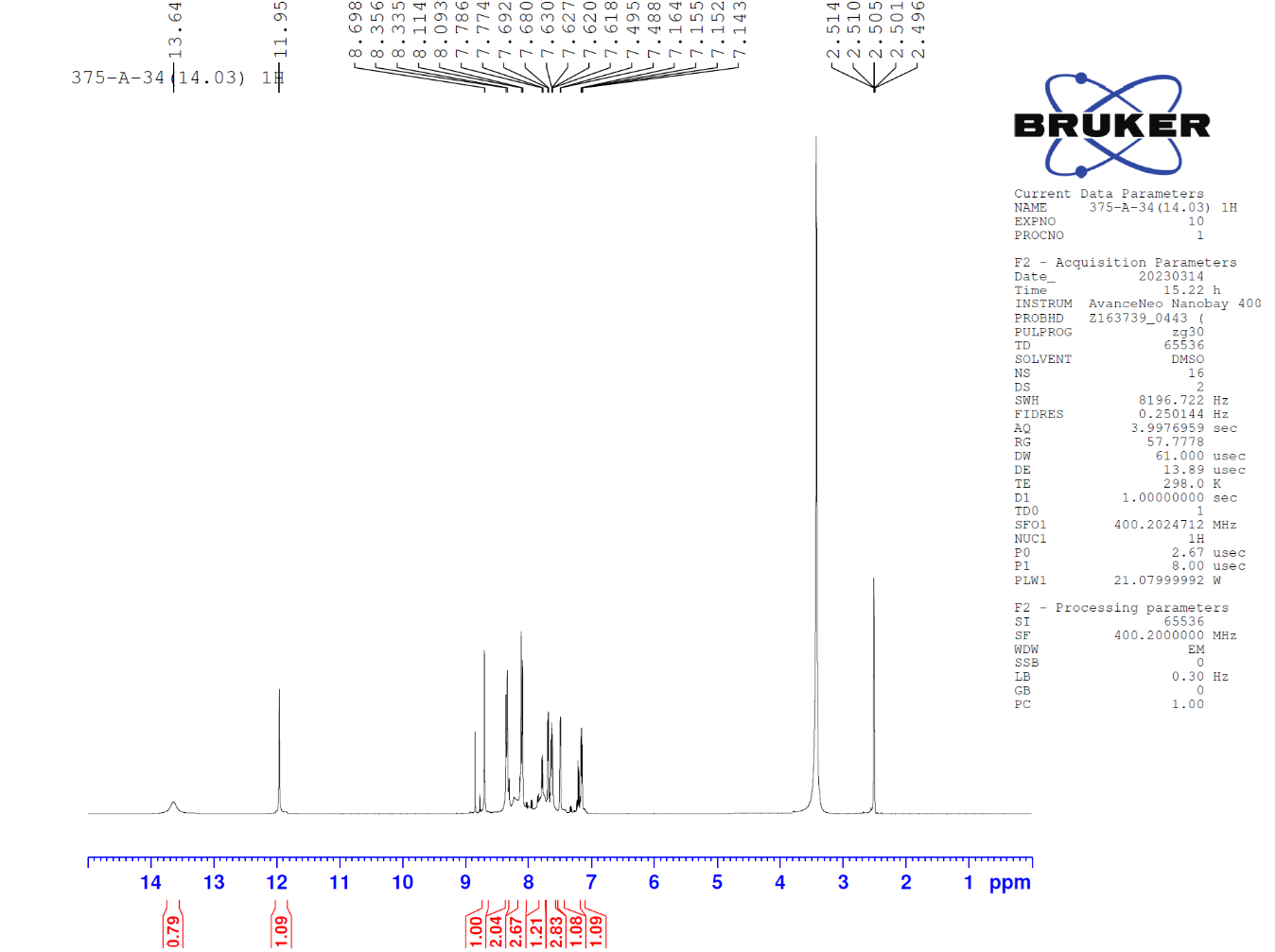
**

**Figure 22.** ^1^H-NMR spectrum of compound **3h**

**
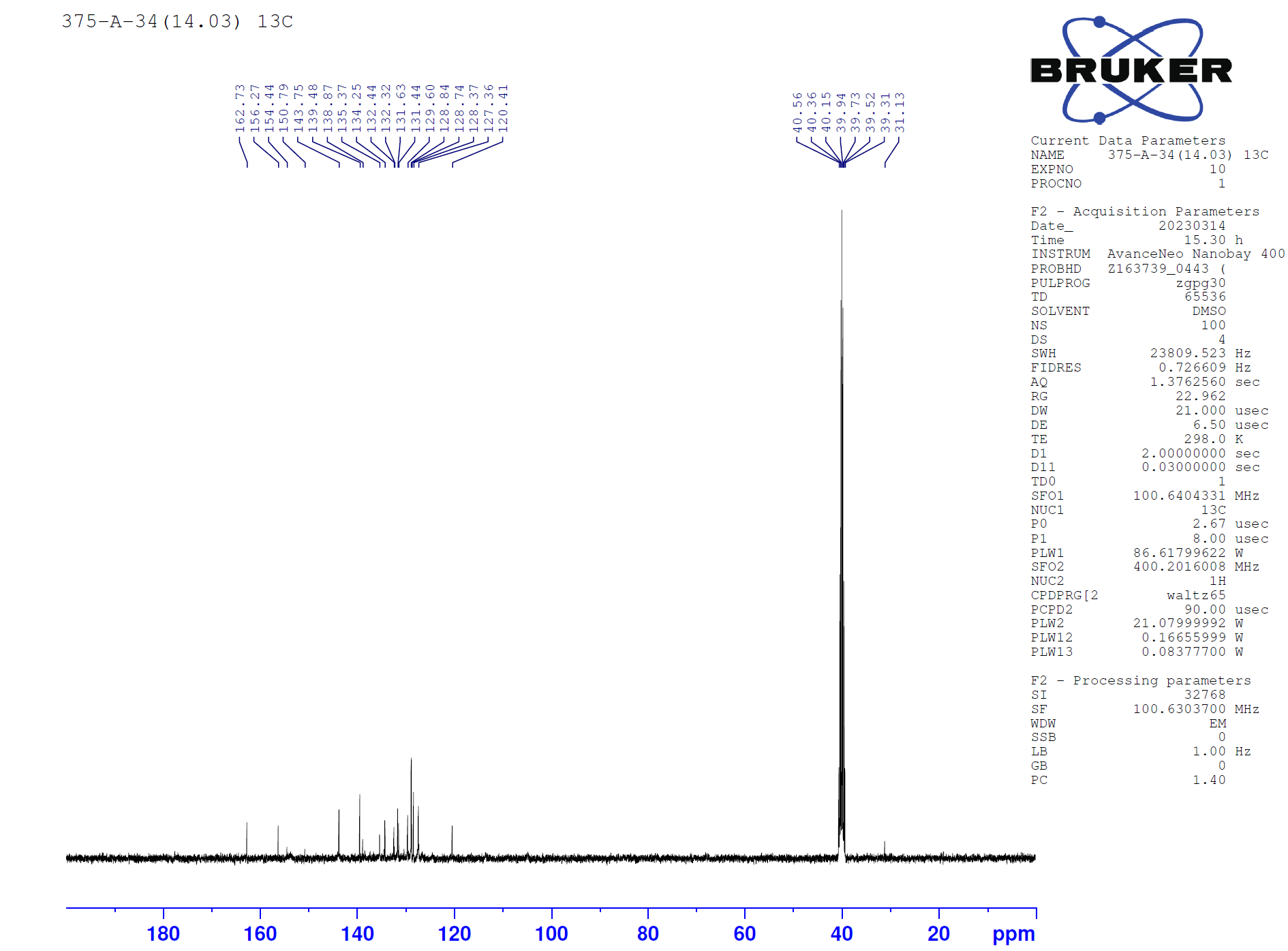
Figure 23.** ^13^C-NMR spectrum of compound **3h**


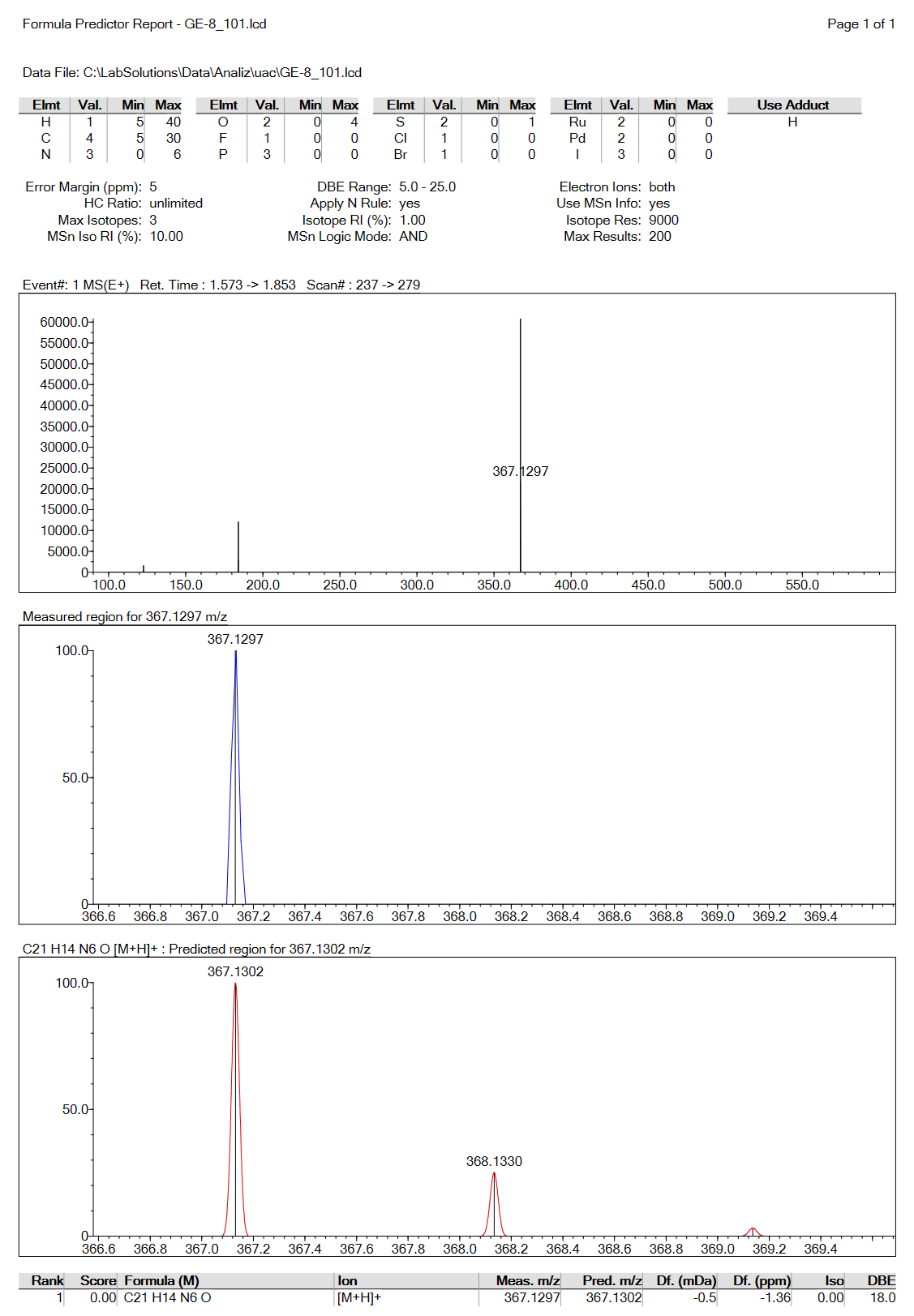


**Figure 24.** Mass spectrum of compound **3h**

**
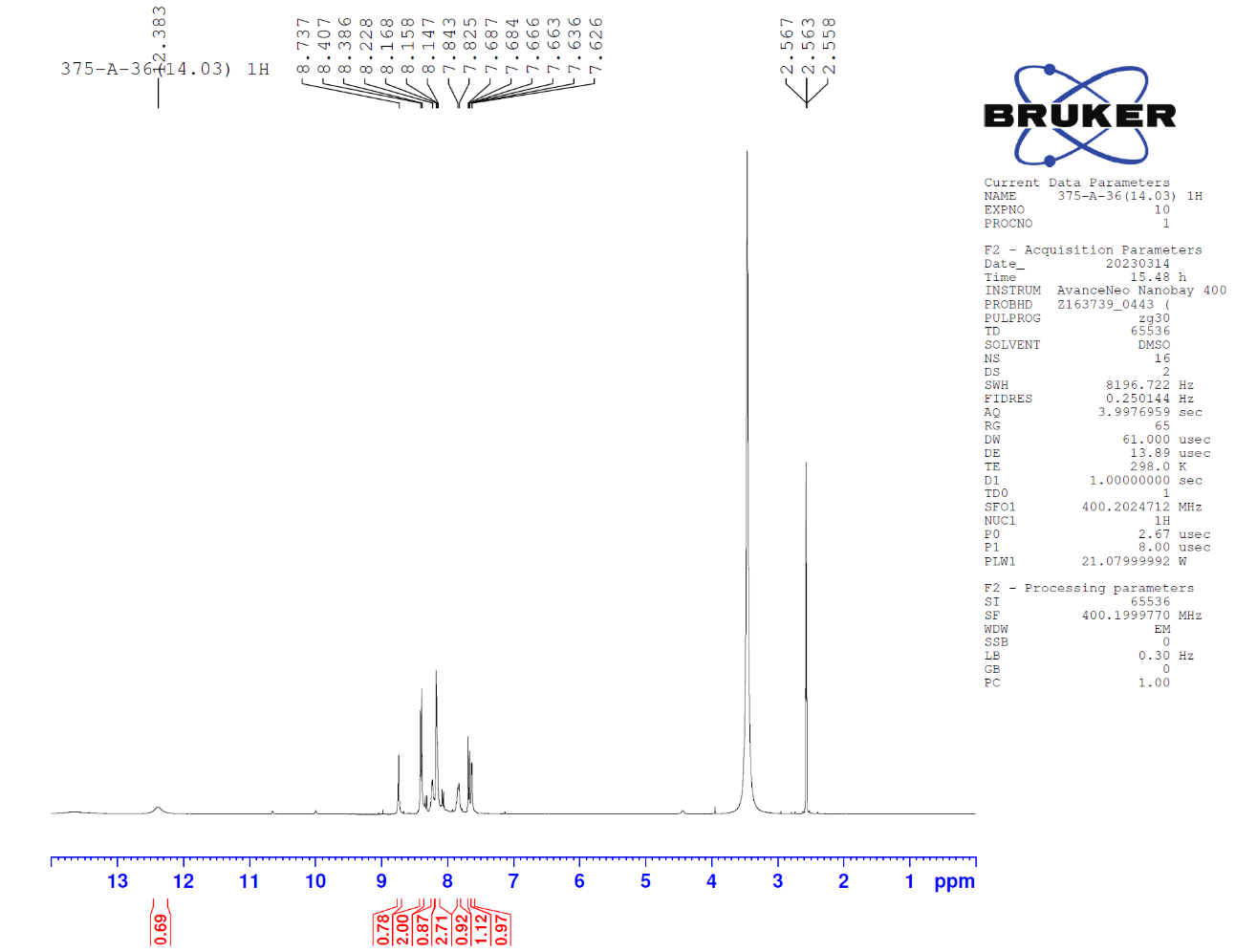
Figure 25.** ^1^H-NMR spectrum of compound **3i**

**
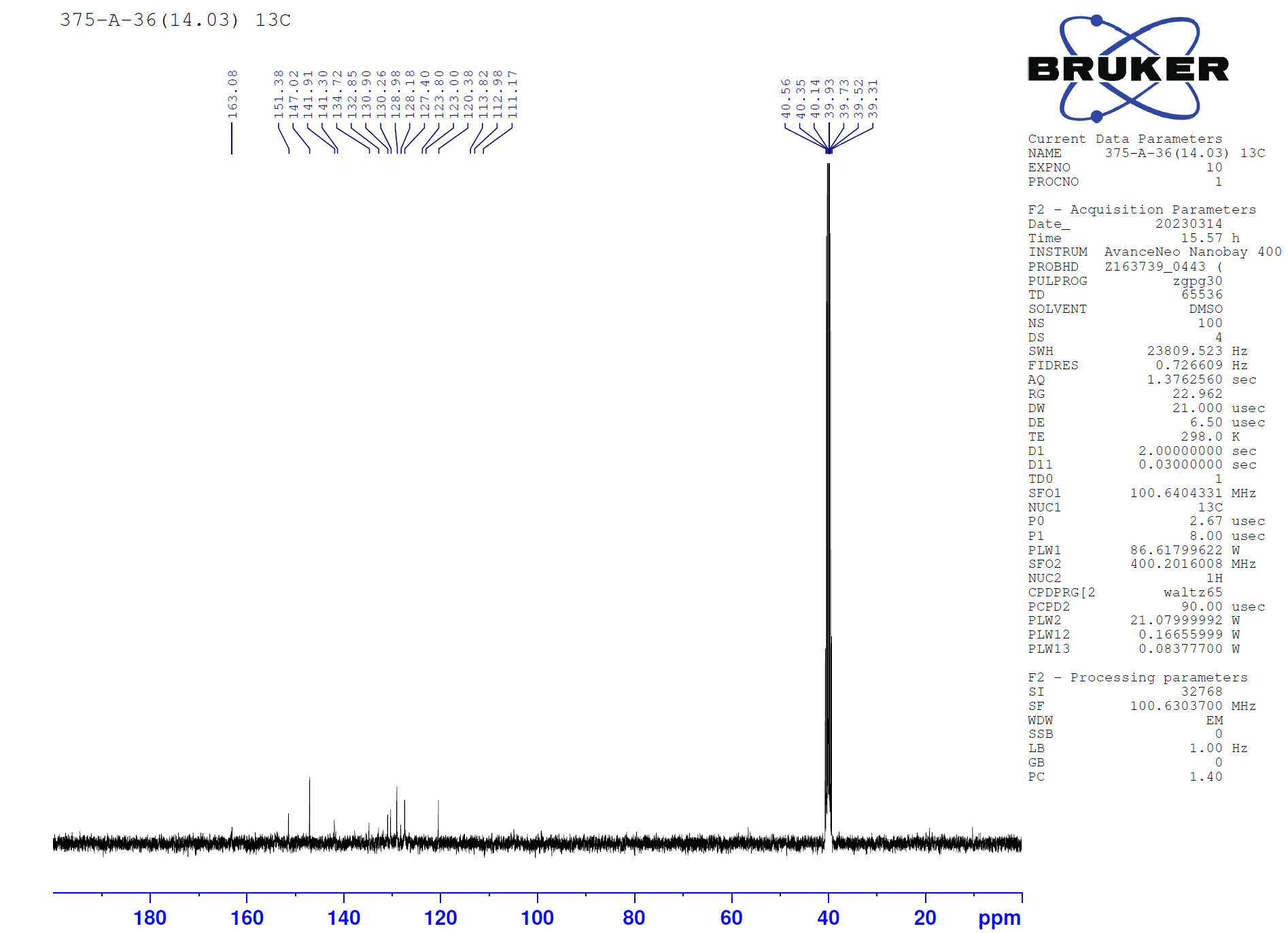
Figure 26.** ^13^C-NMR spectrum of compound **3i**


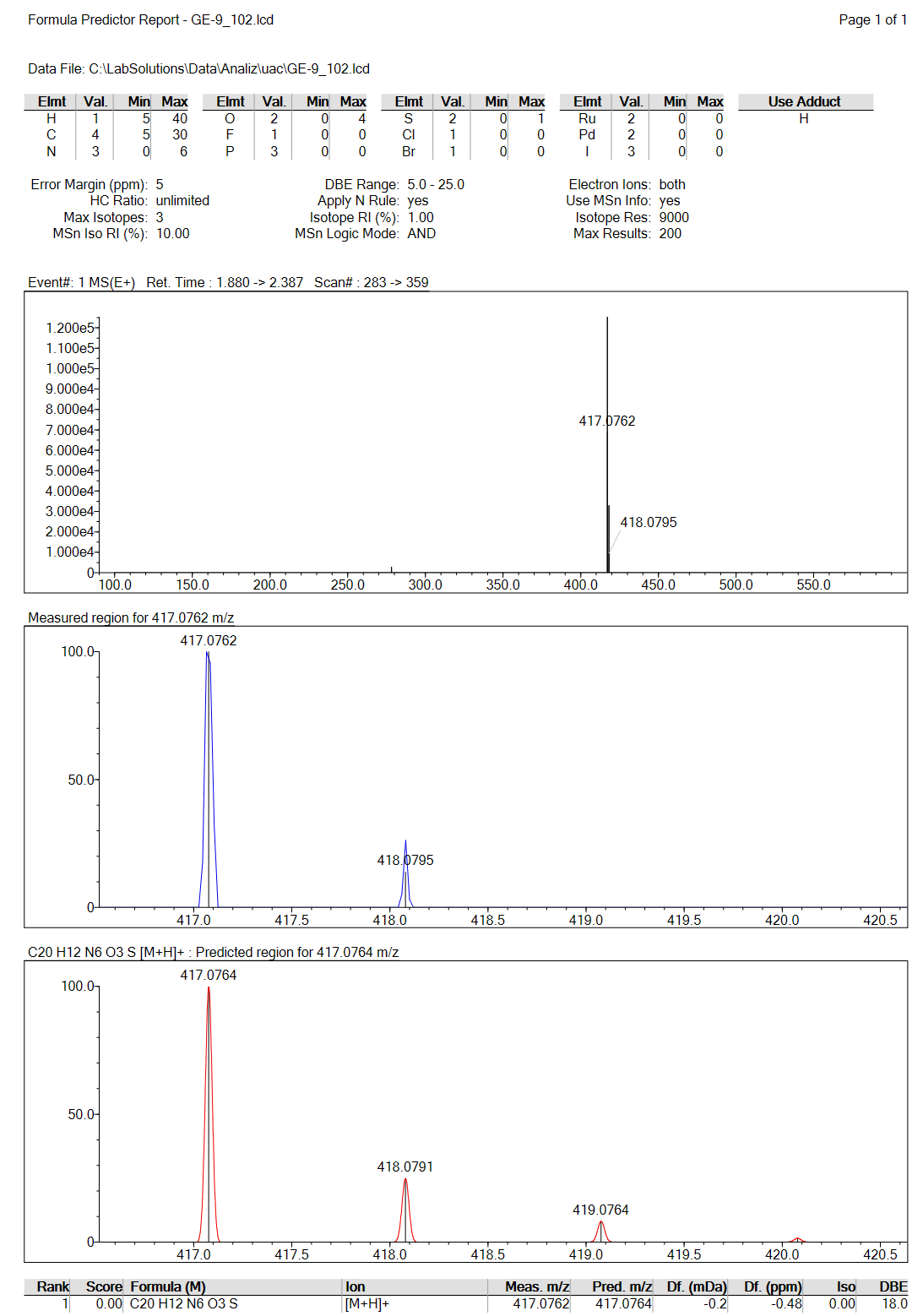


**Figure 27.** Mass spectrum of compound **3i**

**
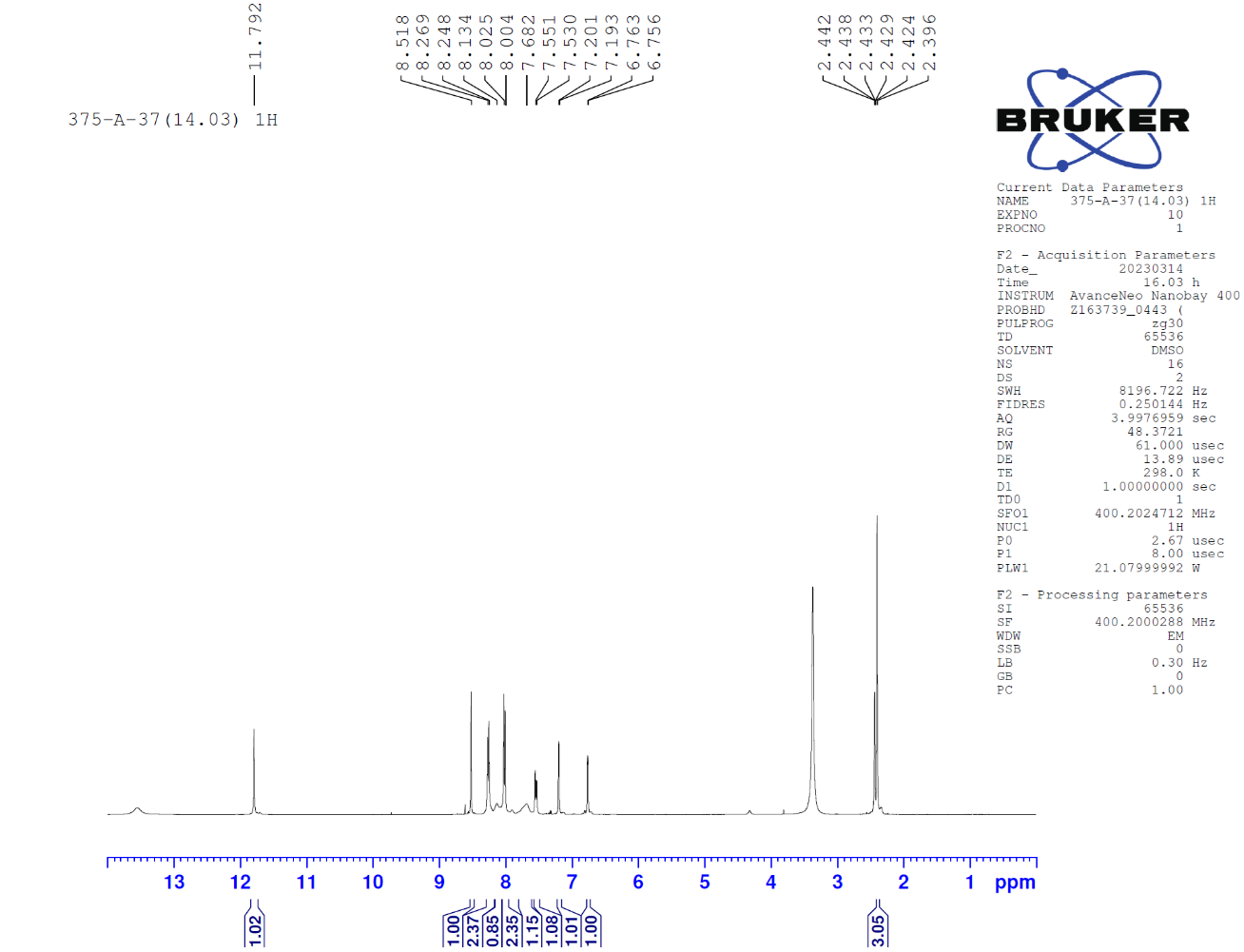
**

**Figure 28.** ^1^H-NMR spectrum of compound **3j**

**
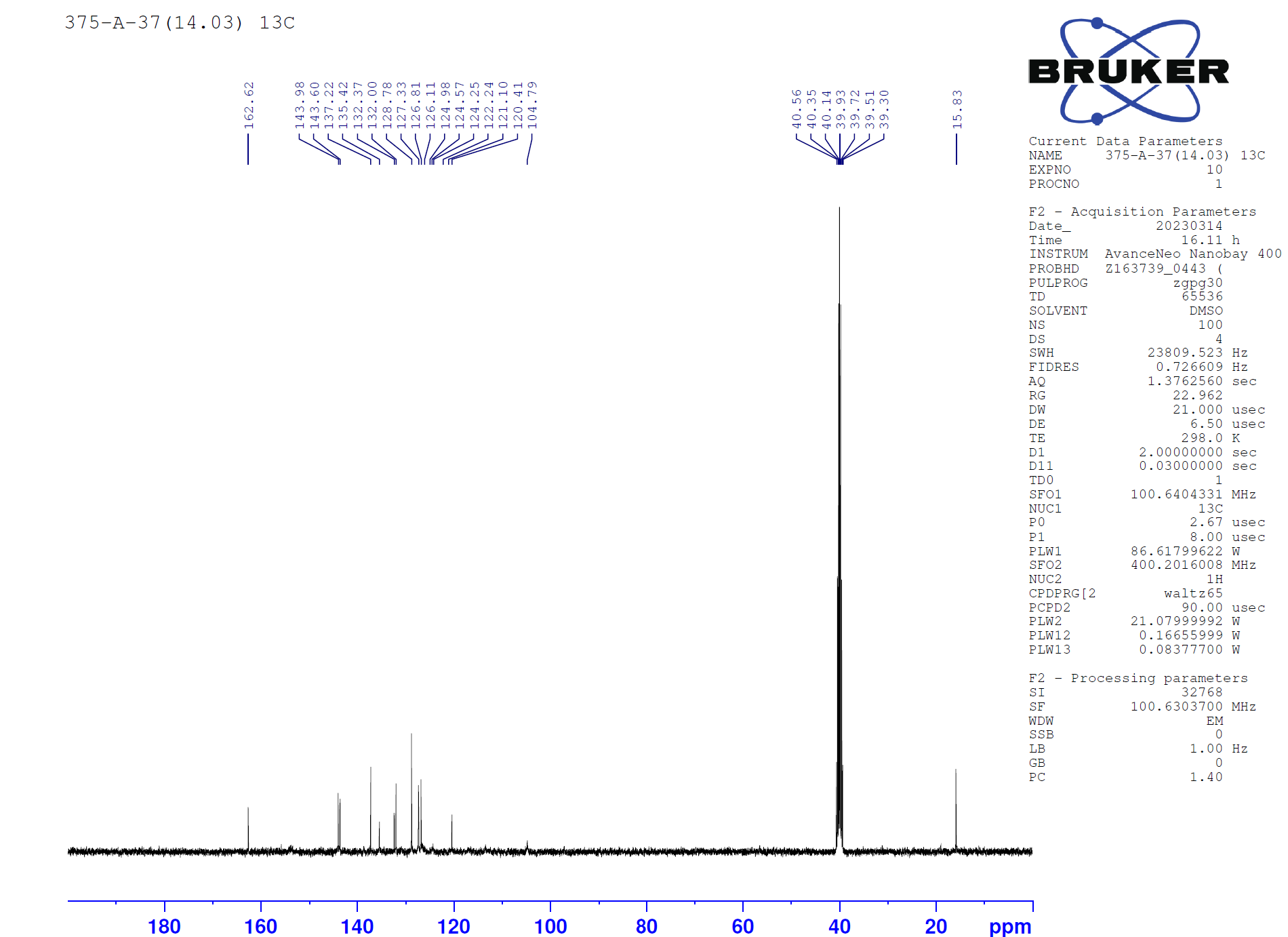
**

**Figure 29.** ^13^C-NMR spectrum of compound **3j**


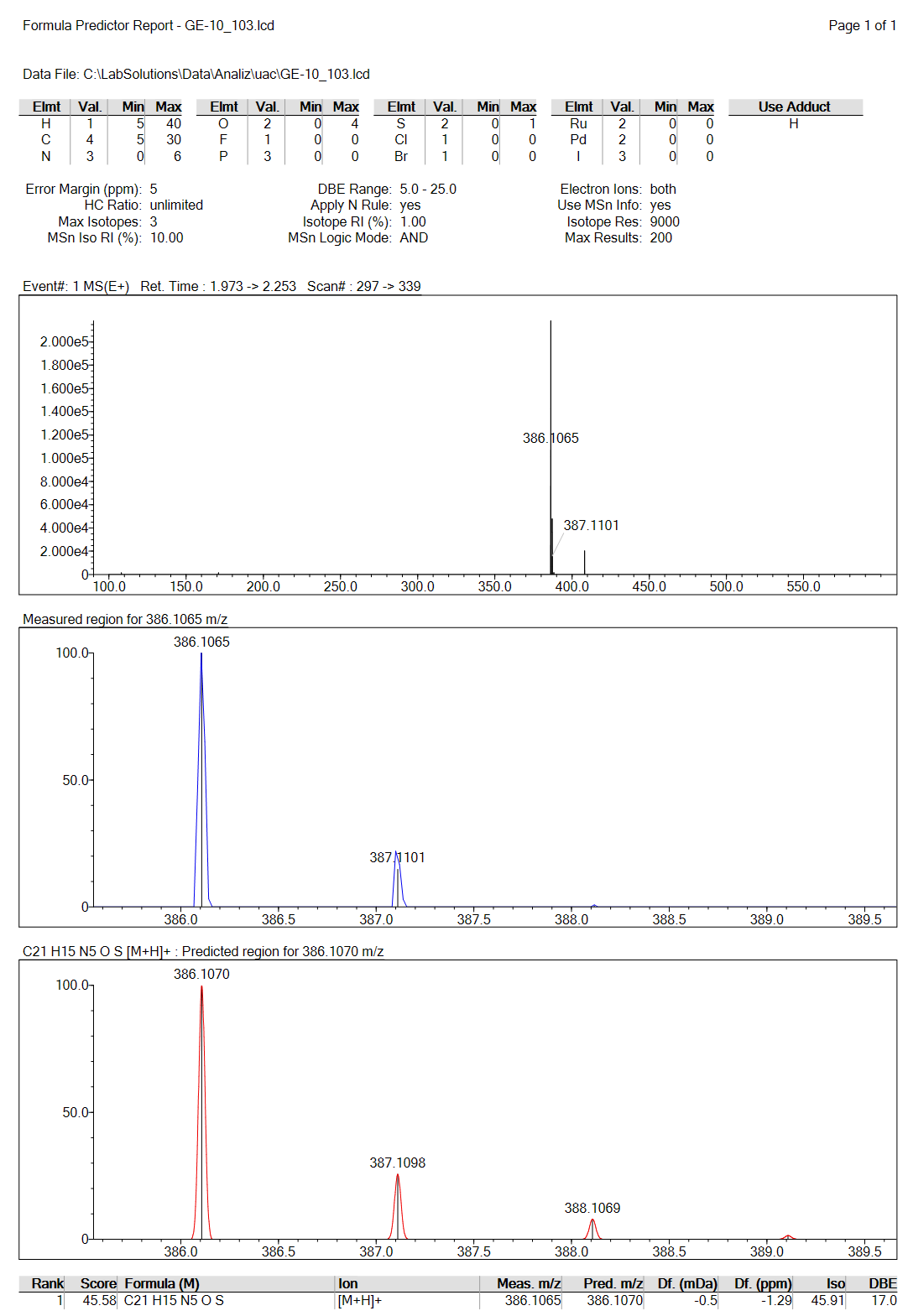


**Figure 30.** Mass spectrum of compound **3j**
